# Expression of matrix metalloproteinases to induce the expression of genes associated with apoptosis during corpus luteum development in bovine

**DOI:** 10.7717/peerj.6344

**Published:** 2019-01-30

**Authors:** Sang Hwan Kim, Ji Hye Lee, Jong Taek Yoon

**Affiliations:** 1 Institute of Genetic Engineering, Hankyong National University, Ansung, Gyeonggi-do, Korea; 2 Major in the Animal Biotechnology, Graduate School of Future Convergence Technology, Hankyong National University, Anseong, Gyeonggi-do, Korea; 3 Department of Animal Life Science, Hankyong National University, Ansung, Gyeonggi-do, Korea

**Keywords:** MMPs, Apoptosis, TIMPs, Bovine, Luteal cell

## Abstract

Here we investigated the expressions of apoptosis-associated genes known to induce programed cell death through mRNA expressions of two matrix metalloproteinases (MMPs) that are involved in the degradation of collagen and basal membrane in luteal cells cultured in the treatment media. Our results show that the activity of MMP-2 gelatinase was higher in the CL2 and CL1 of luteal phase, was gradually decreased in the CH2 and CH3 of luteal phase. In particular, the expressions of P4-r and survival-associated genes (IGFr, PI3K, AKT, and mTOR) were strongly induced during CL3 stage, whereas the levels of these genes in corpus luteum (CL) were lower during CL2 and CL1 stages. In the cultured lutein cells analyzed, we found that as MMPs increase, genes related to apoptosis (20α-hydroxy steroid dehydrogenase and caspase-3) also increase. In other words, the results for P4-r and survival-related gene expression patterns in the luteal cells were contrary to the MMPs activation results. These results indicate that active MMPs are differentially expressed to induce the expression of genes associated with programed cell death from the degrading luteal cells. Therefore, our results suggest that the MMPs activation may lead to luteal cell development or death.

## Introduction

The hormone-controlled metabolic mechanism of corpus luteum (CL) causes physiological changes in embryo implantation, and abnormal regression in CL may cause early abortion, owing to the atretic follicle and decrease in the progesterone metabolic process (Shi & Segaloff, 1995; Lei, Chegini & Rao, 1991). In particular, the main cause of abnormal CL is associated with the apoptosis by matrix metalloproteinases (MMPs) during CL regression in the ovarian tissues. During the development of CL in the ovary, structural reorganization is achieved through the degradation of type VI collagen the main ingredient of the basement membrane (Kim et al., 2014a). This process is influenced by two typical degradation enzymes, MMP-2 and MMP-9 (Visse & Nagase, 2003), and accompanied with changes in the components and structures of extracellular matrix (ECM) (Greenwald & Roy, 1994). Thus, the CL remodeling process is mediated by several ECM proteases such as MMPs and plasminogen activator system (Smith et al., 1999; Woessner, 2002). The luteinizing hormone (LH) level surges during the formation of CL, wherein it synchronizes a series of biochemical events such as increase in progesterone, prostaglandins, and different growth and angiogenic factors (Curry & Osteen, 2003). The angiogenic factors basic fibroblast growth factor and vascular endothelial growth factor (VEGF) have been reported to induce MMP expression in endothelial cells of CL tissues (Partridge, Hawker & Forough, 2000; Sato et al., 2000). Both of these factors are upregulated after LH surge in mature bovine follicles and in early CL (Berisha et al., 2000a, 2000b, 2006a, 2006b). In addition, it has been reported that 20α-hydroxy steroid dehydrogenase (20α-HSD) converts progesterone to inactive 20a-hydroxypregn-4-en-3-one (20α-OHP) under the control of prolactin and controls the function of the CL (Shizuo et al., 1989). MMPs are involved in the regulation of CL activation and inhibition. It has been reported that MMP-1, -2, -9, and -14 levels increase drastically in the bovine CL tissue during luteolysis induced by prostaglandin F2α (PGF2α) injection (Kliem et al., 2007). As luteal cells have been shown as the source of MMPs in the luteum during luteolysis (Kliem et al., 2007), MMPs secreted by luteal cells may breakdown the ECM surrounding them in a manner in which the MMPs secreted by cancer cells breakdown the ECM in metastasis (Sugino & Okuda, 2007). In other words, luteal cells undergoing continual structural changes activate MMPs during development, resulting in the degradation of CL tissue. To test this hypothesis, we investigated the expression of caspase-3 (Casp-3) and 20α-HSD known to be involved in the programed cell death caused by mRNA expressions of two MMPs that degrade the collagen and basal membrane in cultured luteal cells.

## Materials and methods

### Collection and preparation of bovine corpus luteal

Ovaries with CLs from Hanwoo (Korean native cattle [age: about 30 months]) were collected at a local abattoir (Pyeong-Nong, Pyeongtaek, South Korea) within 1 h from exsanguination. The samples were placed into an LN2 freezer box and were transported to the laboratory within 2 h. In short, for the determination of the CL phase, a microscope evaluation was taken into account, which evaluated the color, consistency, and uterus of the entire ovaries. Estrous cycle luteal stages were classified into five categories by macroscopic observation as follows: corpora hemorrhagic (CH2) (1–2 cm), CH3 (>2 cm), CL3 (2.1–2.8 cm), CL2 (2.3–2.5 cm), CL1 (>2 cm) (Lee et al., 2015) (Table 1). This study was carried out in strict accordance with the recommendations in the Guide for the Care and Use of Laboratory Animals of the National Institutes of Health. The protocol was approved by the Committee on the Ethics of Animal Experiments of the Hankyong National University (Permit Number: 2016-1).

**Table 1:** Morphological criteria for the classification of luteum based on the days of the bovine estrous cycle.

Criteria	Developmental stage of corpus luteum				
	CH2	CH3	CL3	CL2	CL1
Surface	Ovulatory point partially covered by epithelial/luteal cells	Covered by luteal tissue	Covered by luteal tissue	Covered by luteal/connective tissue	Covered by connective tissue
Color of luteum	Brownish red	Reddish brown	Tan to orange	Orange to yellow	Light yellow to white
Diameter (cm)	1.1–1.6	1.8–2.0	2.1–2.8	2.3–2.5	1.7–2.0

**Note:**

Lee et al. (2015).

### Isolation and culture of luteal cell

Luteal cells were obtained as per the method of O’Shaughnessy & Wathes (1985). The CL, collected from the cow’s ovary, separated using collagen decomposition and collected cells (pellet) with centrifuges (5 min at 50 x g). Luteal cells were plated in Dulbecco’s modified Eagle’s medium (DMEM)/Nutrient Mixture F-12 Ham (D/F, 1:1, v/v; no. D8900; Sigma-Aldrich, St. Louis, MO, USA) containing 10% fetal bovine serum (FBS; Life Technologies, cat no. 16000-044) and 20 μg/mL gentamicin (cat no. 15750-060; Life Technologies) and cultured with 5% CO_2_ in air at a density of 2 ± 0.3 × 10^6^ cells in a T-25 tissue culture flask (Becton Dickinson and Co., Franklin Lakes, NJ, USA) for 16 h to facilitate cell attachment. Cell viability was greater than 80%, as assessed by trypan blue exclusion assay. Luteal cells were cultured in treatment culture medium (DMEM with Lutalyse, Dinoprost tromethamine, Pfizer, Puurs, Belgium; The final 0.5 IU/mL was used in the experiment.) for 24, 48, 72, or 96 h at 37 °C in a humidified atmosphere containing 5% CO_2_ for analysis (Nelson et al., 1992). The cells were removed from the culture flask and subjected to centrifugation. The pellet was resuspended in one mL fresh DMEM. An aliquot of the cell suspension was stained by mixing with 0.4% trypan blue solution (1:1), and 10 uL of the cell mixture was pipetted onto a counting slide. TC10 automated cell counter (Bio-Rad Laboratories, Inc., Hercules, CA, USA) was used to determine the density of live cells and evaluate the total cells in the sample.

### Expression of live and MMP-associated genes

Total RNA was extracted from CL tissues (estrous cycle luteum) and cultured cells using TRIzol reagent (Invitrogen, Grand Island, NY, USA). Total cDNA for analysis was synthesized by reverse transcription of mRNA (1.0 µg) using an SuperScripttm II Reverse Transcriptase (Invitrogen, Grand Island, NY, USA). A total of one µL cDNA was added to SYBR Green (TOYOBO, Tokyo, Japan) mixture and real-time polymerase chain reaction (RT-PCR) was performed using specific gene primers (Table 2) at an annealing temperature of 60–65 °C for 30 cycles. Data were analyzed using line-gene K program (Bioneer Technology, Tokyo, Japan). Each PCR results were converted to fold increase according to the semi-log amplification plot of the geometric region.

**Table 2:** Primer for real-time PCR analysis of apoptosis-associated genes.

Primer name	Sequence	Gene ID
Apoptosis associated genes		
Bos 20α-HSD FW	5′ GGA AAG CGG ATA GTC AGG GTG ATC 3′	NM_001167660.1
Bos 20α-HSD RV	5′ GCC ATT GCC AAA AAG CAC AAG 3′	
Bos Casp-3 FW	5′ AGC CAT GGT GAA GAA GGA ATC A 3′	NM_001077840.1
Bos Casp-3 RV	5′ GGT ACT TTG AGT TTC GCC AGG A 3′	
Survival associated genes		
Bos mTOR FW	5′ TCT CAT GGG TTT TGG AAC GA 3′	XM_005216989.1
Bos mTOR RV	5′ TGA GAG CTG TAC CCC AGC AG 3′	
Bos PCNA FW	5′ GCA CTG AGG TAC CTG AAC TT 3′	NM_001034494.1
Bos PCNA RV	5′ TCT TCA TCC TCG ATC TTG GG 3′	
Bos IGF-r1 FW	5′ CGT GTG GGT GCA TTT CTG TTA C 3′	NM_001244612.1
Bos IGF-r1 RV	5′ GCC TCA CCT CCT TAT TCC ATT G 3′	
Bos PI3K FW	5′ CAG CAA AAC TAC TGC TTA TTC TTC	NM_174575.1
Bos PI3K RV	5′ GCC TCA CCT CCT TAT TCC ATT G 3′	
Bos AKT FW	5′ CCA GAT GAT CAC CAT TAC GC 3′	NM_001191309.1
Bos AKT RV	5′ CAA ACG CAT CCA GAA ATA AAA A 3′	
Bos VEGF FW	5′ TGT AAT GAC GAA AGT CTG CAG 3′	NM_174216.1
Bos VEGF RV	5′ TCA CCG CCT CGG CTT GTC ACA 3′	
Bos PAPP-A FW	5′ CGC CCA AAC GGT CAA AGA CT 3′	XM_002689953.3
Bos PAPP-A RV	5′ GGG AGA TTC CTG GTG CAG TA 3′	
Bos P4-r FW	5′ TGG TTT GAG GCA AAA AGG AG 3′	NM_001205356.1
Bos P4-r RV	5′ CCC GGG ACT GGA TAA ATG T 3′	

### Extraction of total protein from the cultured cells and corpus luteum of bovine

For zymography and enzyme-linked immunosorbent assay (ELISA), protein was extracted from CL tissue and lutein cells using the Pro-prep solution (Intron, Seoul, Korea), according to the manufacturer’s instruction. The final protein samples were stored at −80 °C.

### Hormone ELISA

For ELISA, protein samples (CL stage: CH2, CH3, CL3, CL2, CL1) were diluted in 100% assay buffer. Target protein (follicle-stimulating hormone [FSH] receptor, LH receptor, and mammalian target of rapamycin [mTOR]) levels were measured using a sandwich ELISA (R&D Systems Europe, Abingdon, UK) according to the manufacturer’s instruction. A primary antibody was applied to a 96-well ELISA plate at 4 °C for 1 day, washed twice using washing buffer (1× PBS with 2.5% Triton X-100), and blocked using 1% skim milk blocking solution at 4 °C for 24 h. After washes with the washing buffer, immune reactions were detected using secondary antibodies (Anti-mouse and rabbit: Abcom, Cambridge, UK) for 2 h, and substrate solution (R&D Systems, Minneapolis, MN, USA) was added for the reaction. To stop the reaction, 1M NH_2_SO_4_ was used, and absorbency was measured at 450 nm.

### Immunohistochemistry

Immunohistochemical detection of MMPs was performed on five µm tissue sections mounted on siliconized slides. First step, paraffin fix tissue sections were deparaffinized with xylene substitute (Polysciences, Warrington, PA, USA) and step by step rehydrated in ethanol (100–70%).

Antigen retrieval was performed by heating the sections at 95 °C in 10 mM sodium citrate (pH 6.0). Endogenous peroxidases were quenched with 0.3% hydrogen peroxide in methanol for 5 min at room temperature (RT). Slides were incubated in a blocking buffer containing 1% goat serum and 3% horse serum in 1× PBS (phosphate-buffered saline) for 1 h at RT. Sample slides were incubated 2 h at RT with antibodies (diluted 1:200 in blocking buffer) against MMP-2 (ab78796; Abcam, Cambridge, UK). Section slides were washed in 1× PBS and then incubated with secondary anti-rabbit, (sc-2054; Santa Cruz Biotechnology Inc., Dallas, TX, USA) antibodies (diluted 1:300 in blocking buffer) for 1 h at RT. Following incubation, section slides were washed and activated for 10 min using the ABC detection kit (Vector, CA, USA); diaminobenzidine (Vector Laboratories, Burlingame, CA, USA) was used as a substrate for HRP. Sections were counterstained with periodic acid-Schiff reagent and Harris’ hematoxylin solution containing 4% acetic acid. Tissues were dehydrated, cleared, and covered with Permount solution (Fisher Chemical, Waltham, MA, USA).

### Immunofluorescence assay

Lutein cells were cultured on sterilized culture slide (SPL, Gyeonggi-do, Korea) and after slides fixed with 4% paraformaldehyde, and blocked with 3% BSA (bovine serum albumin) in 1× PBS. Dehydration and permeabilization were performed by freezing the slides at −20 °C in five mM 0.1% Triton X-100 in PBS. After blocked with 3% BSA in 1× PBS, sample slides were incubated with the monoclonal antibody against the active form of MMP-2, MMP-9 (Santa Cruz Biotechnology Inc., Dallas, TX, USA), TIMP-2 (sc-9905, Santa Cruz Biotechnology Inc., Dallas, TX, USA), and TIMP-3 (sc-6836, Santa Cruz Biotechnology Inc., Dallas, TX, USA) at 1:150 dilutions. After washing, the slides were incubated with anti-rabbit and mouse IgG conjugated to Alexa Fluor 488 or Alexa Fluor 594 (Molecular Probes). After counterstained with one g/mL Hoechst 33258 solution, and slides were mounted using fluorescent mounting medium (Dako, Carpinteria, CA, USA). Detected protein images were analyzed using Olympus AX70 fluorescence microscope fitted.

### Zymography

To gelatinases-activity analyzed of MMPs, 20 mg of total proteins were mixed with two µL FOZ loading buffer (5% bromophenol blue, 10% sodium dodecyl sulfate [SDS], and 2% glycerol in distilled water) on ice for 5 min. The sample was subjected to SDS polyacrylamide gel electrophoresis using gels containing 100 mg/mL gelatin A and B for 90 min at 150 V. After electrophoresis, the proteins were renatured twice for 20 min in a renaturation buffer (2.5% Triton X-100 in 1× PBS) and the gel was washed with sterile water for 20 min. After renaturation, samples were incubated in zymography reaction buffer (1M Tris–HCl, 5M sodium chloride [NaCl], 1M calcium chloride [CaCl_2_], 0.2 mM zinc chloride [ZnCl_2_], 0.2% Triton X-100, and 0.02% sodium azide [NaN_3_] in 1× PBS; pH 7.5) at 37 °C for 18 h. The gel was stained with Coomassie Brilliant Blue for 1 h to reveal clear areas of digested gelatin (Kim et al., 2014b).

### Statistical analysis

Data were subjected to *t*-test analysis and general linear model and Duncan’s multiple range tests using Statistical Analysis System software (SAS Institute, version 9.4, Cary, NC, USA). The statistical significance was established at *p* < 0.05.

## Results

### Expression of mTOR protein during the development of corpus luteum

**Figure 1:**
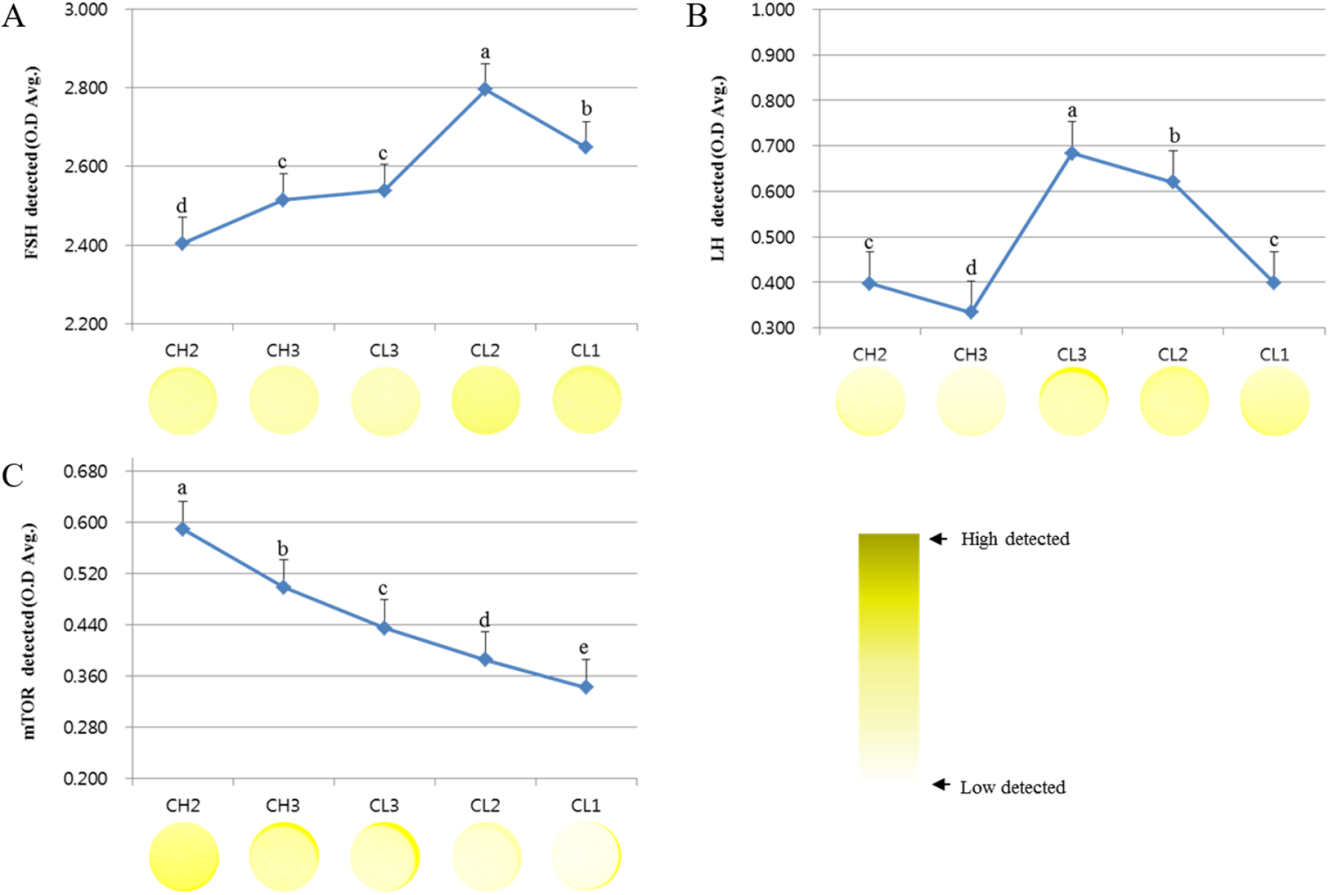
ELISA analysis of FSH, LH, and mTOR proteins at different stages of bovine corpus luteum. ELISA experiments were repeated thrice, and data are average fold change (mean ± SD). The darker the yellow color, higher is the expression. (A) FSH protein level. (B) LH protein level. (C) mTOR protein level.^a,b,c,d,e^ Different letters within the same column represent a significant difference (*p* < 0.05).

We analyzed the survival signal-associated factors during the development of CL (Fig. 1). The concentrations of FSH receptor (optical density [O.D.] values) analyzed during the development of CL were as follows: CH2, 2.404 ± 0.054; CH3, 2.515 ± 0.015; CL3, 2.539 ± 0.021; CL2, 2.795 ± 0.27; and CL1, 2.648 ± 0.017. These values tended to increase from CH2 to CL2 stage and were lower at CL1 stage. On the other hand, the expression of LH receptor decreased from CH2 (0.397 ± 0.012) to CH3 (0.334 ± 0.008) stage but was maximum at CL3 stage (0.684 ± 0.022), followed by a decrease from CL3 to CL1 (0.398 ± 0.014) stage (*p* < 0.05). The level of mTOR protein during the development of CL was higher during CH2 stage (0.589 ± 0.012) but decreased from CH3 (0.474 ± 0.023) to CL1 (0.342 ± 0.015) stage. Overall, the expression of LH receptor was more readily induced during CL stages than during CH stages. The expression of FSH receptor was higher during CL2 and CL1 stages than during other stages.

### Survival-associated gene expression at CL stage

**Figure 2:**
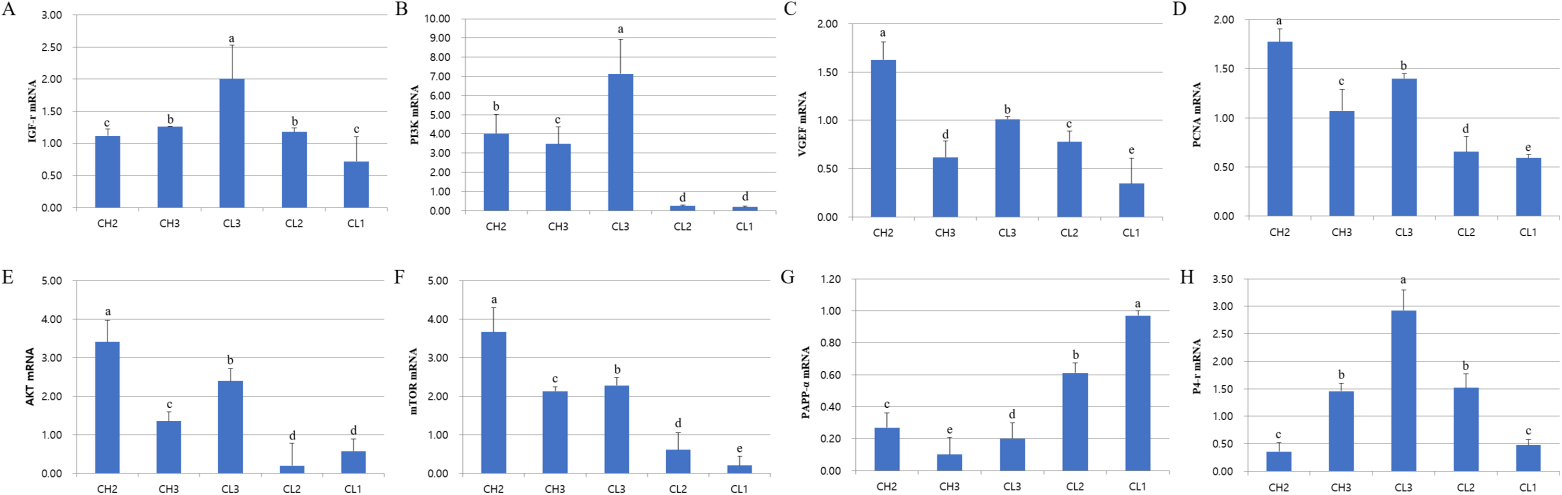
Expression of life signal-associated mRNAs in the corpus luteum cells. Experiments were repeated thrice, and data are expressed as mean ± SD. Total RNA was extracted and analyzed by real-time PCR. (A) IGF-r mRNA level. (B) P13K mRNA level. (C) VEGF mRNA level. (D) PCNA mRNA level. (E) AKT mRNA level. (F) mTOR mRNA level. (G) PAPP-a mRNA level. (H) P4-r mRNA level.^a,b,c,d,e^ Significant difference (*p* < 0.05).

The expression of genes associated with cell development (IGFr[insulin grows factor-receptor], PI3K[Phosphoinositide 3-kinase], AKT, and mTOR), cell activity (VEGF and PCNA[Proliferating cell nuclear antigen]), and hormones (LH, PAPP-a[pregnancy-associated plasma protein A] and P4-r[progesterone-receptor]) was evaluated at each stage. The expressions of these mRNAs were different at each CL stage (Fig. 2). RT-PCR analysis results revealed that the expressions of IGFr, PI3K, and P4-r were higher during CL3 stage than in CH stages. On the other hand, the expressions of VEGF, PCNA, and AKT were higher during CH stages than in CL stages; PAPP-a expression was maximum during CL1 stage and decreased from CL1 to CH3 stage (*p* < 0.05). The expression of all genes except PAPP-a was low from CL2 to CL1 stage, whereas the levels of AKT and mTOR mRNAs at CL3 stage were similar to those at CH2 stage. To summarize, the expressions of P4-r and survival-associated genes (IGFr, PI3K, AKT, and mTOR) were strongly induced during CL3 stage, whereas the mRNA levels of these genes in CL were lower during CL2 and CL1 stages (*p* < 0.05).

### Changes in gelatinase activation at luteal stage

**Figure 3:**
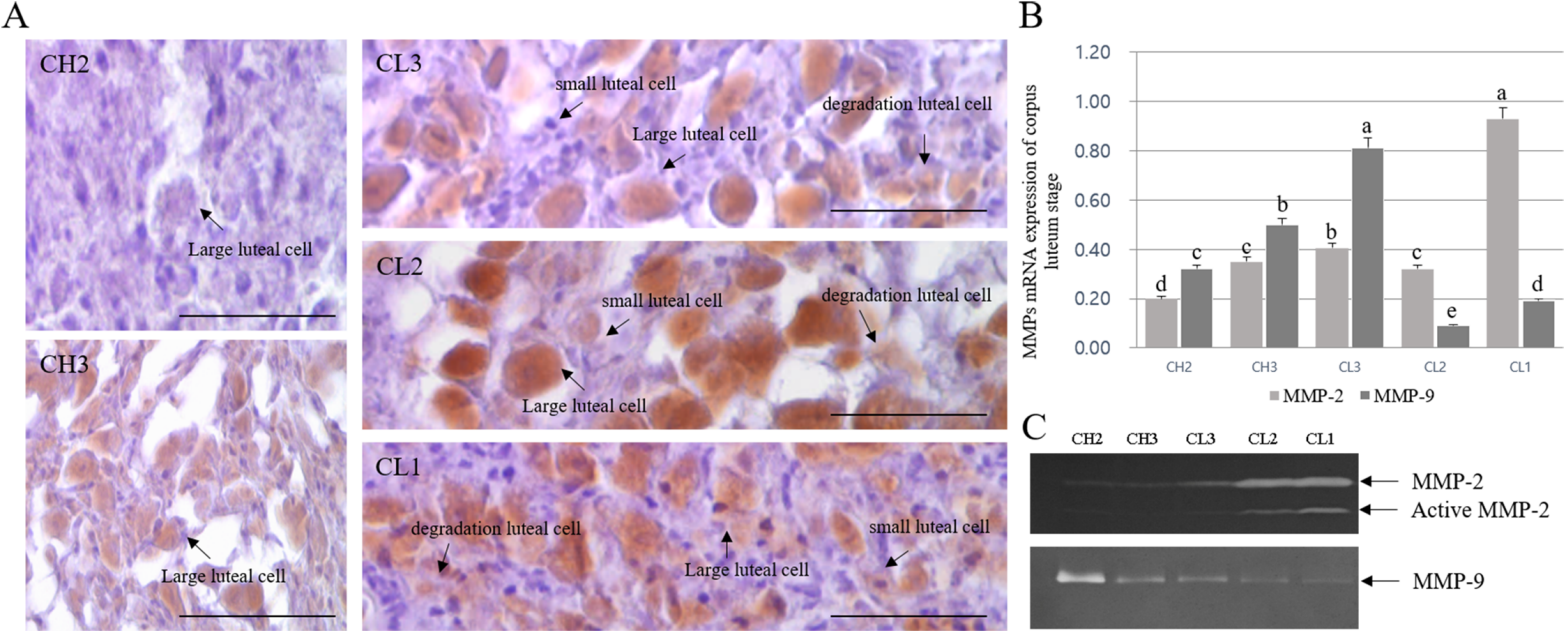
Immuno-detection and expression analysis of MMP proteins at different stages of bovine corpus luteum. Brown color indicates cells positive for MMP, and black arrows indicate type of cell. Corpus luteum cells were counterstained with hematoxylin. A (large figure); magnification ×400. (A) Immunohistochemistry of MMP-2 protein. (B) Real-time PCR. (C) Zymography.^a,b,c,d,e^ Different letters within the same column represent a significant difference (*p* < 0.05).

The expression and activation patterns and localization of MMPs during different stages of CL are shown in Fig. 3. MMP-2 localization was mostly detected in large luteal cells, whereas it was mostly absent in small luteal cells. In addition, MMP-2 expression was low in degrading luteal cells. Although the expression of MMP-2 increased from CH2 to CL2 stage, MMP-2 protein level in large luteal cells of CL3 stage was higher than expected. The expression decreased in large luteal cells of CL1 stage (Fig. 3A). The expression of MMPs during different CL stages was in line with the results of MMP localization, as evident from RT-PCR analysis. In particular, levels of MMP-9 were elevated during CL3 as compared to other stages and were mostly higher during CL1 stage (Fig. 3B). The activity of gelatinase in CL of bovine was evaluated using zymography (Fig. 3C). In general, the activity of gelatinase was higher during CL stage than during CH stage. From CH2 to CL3 stage, the activity of MMP-2 gelatinase was similar during CL stages, but MMP-2 level was higher from CL2 to CL1. However, MMP-9 level decreased from CH2 to CL1.

### Immuno-detection and mRNA expression of MMPs and apoptotic genes in luteal cells cultured from 24 to 96 h

**Figure 4:**
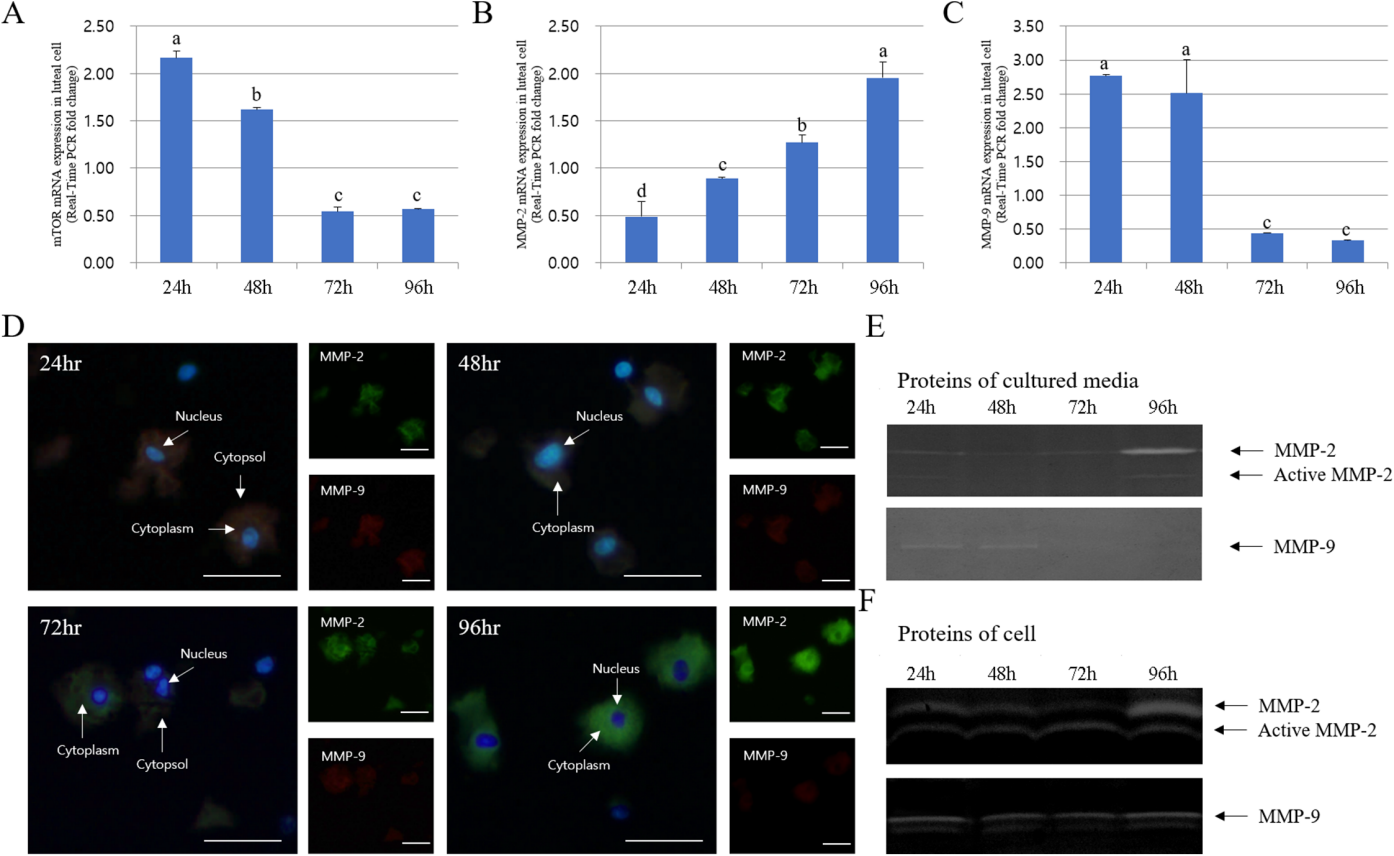
Immunofluorescence and expression analysis of MMPs at various stages of corpus luteum from 24 to 96 h in cultures. White arrows indicate cells. Blue fluorescent indicates nucleus, while green fluorescent indicates MMP-2, red fluorescent indicates MMP-9. Cells were counterstained with Hoechst 33258. Large figure, magnification ×400; small figure, magnification ×200. (A) mTOR mRNA level. (B) MMP-2 mRNA level. (C) MMP-9 mRNA level. (D) Immunofluorescence. (E) Zymography analysis of cultured medium. (F) Zymography analysis of protein.^a,b,c,d^ Different letters within the same column represent a significant difference (*p* < 0.05).

The expression of MMPs in cultured luteal cells followed a pattern similar to that observed for MMP localization. The protein expression patterns in cultured luteal cells in the presence of PGF2α are shown in Fig. 4. MMP and TIMP protein expression were mostly detected in the cytoplasm of luteal cells and were maximum from 24 to 96 h. MMP-2 protein expression mostly increased from 24 to 96 h; however, MMP-9 expression markedly decreased from 24 to 96 h. During the developmental stages, more MMP-2 was expressed in cells cultured for 96 h than in those cultured for 24 h. In cultured cells, MMP-9 expression was increased in the cytoplasm of luteal cells at 24 h but decreased at 96 h (*p* < 0.05).

**Figure 5:**
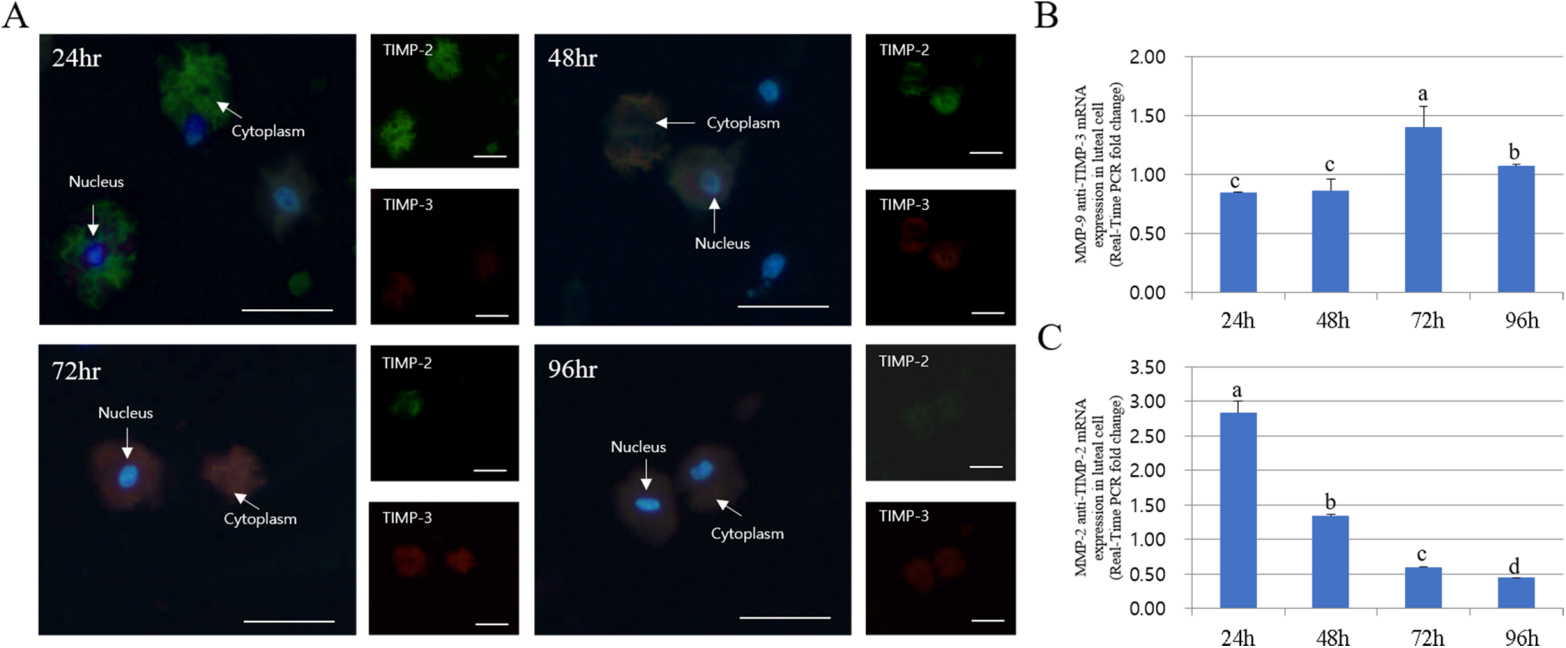
Immunofluorescence and expression analysis of TIMPs at different stages of corpus luteum from 24 to 96 h in cultures. White arrows indicate cells. Blue fluorescent indicates nucleus, while green fluorescent indicates TIMP-2, red fluorescent indicates TIMP-3. Cells were counterstained with Hoechst 33258. Large figure, magnification ×400 and small figure, magnification ×200. (A) Immunofluorescence. (B) TIMP-3 mRNA level. (C) TIMP-2 mRNA level.^a,b,c,d^ Different letters within the same column represent a significant difference (*p* < 0.05).

The expression pattern of MMPs and TIMPs was strikingly different. The expression of TIMP-2 (MMP-2 inhibitor) was low at both 72 and 96 h in the cytoplasm of all cells. Thus, TIMP-2 protein expression was higher at early developmental stages. TIMP-2 was expressed at higher levels in cells cultured for 24 h than those cultured for 94 h, whereas its expression in the cytoplasm was higher in cells cultured for 48 h. However, TIMP-3 (MMP-9 inhibitor) expression increased from 24 to 72 h in luteal cells and decreased at 94 h (*p* < 0.05). In particular, TIMP-3 expression pattern was high in cells cultured for 72 h but was lower than the expression of TIMP-2. RT-PCR results for MMP and TIMP expression in cultured luteal cells were similar to the results of immuno-detection (Fig. 5).

The expression of mTOR, MMP-9, and TIMP-2 decreased from 24 to 96 h in cultured luteal cells, whereas that of MMP-2 and TIMP-3 increased. MMP activation patterns observed during zymography were similar to those observed with other experiments.

**Figure 6:**
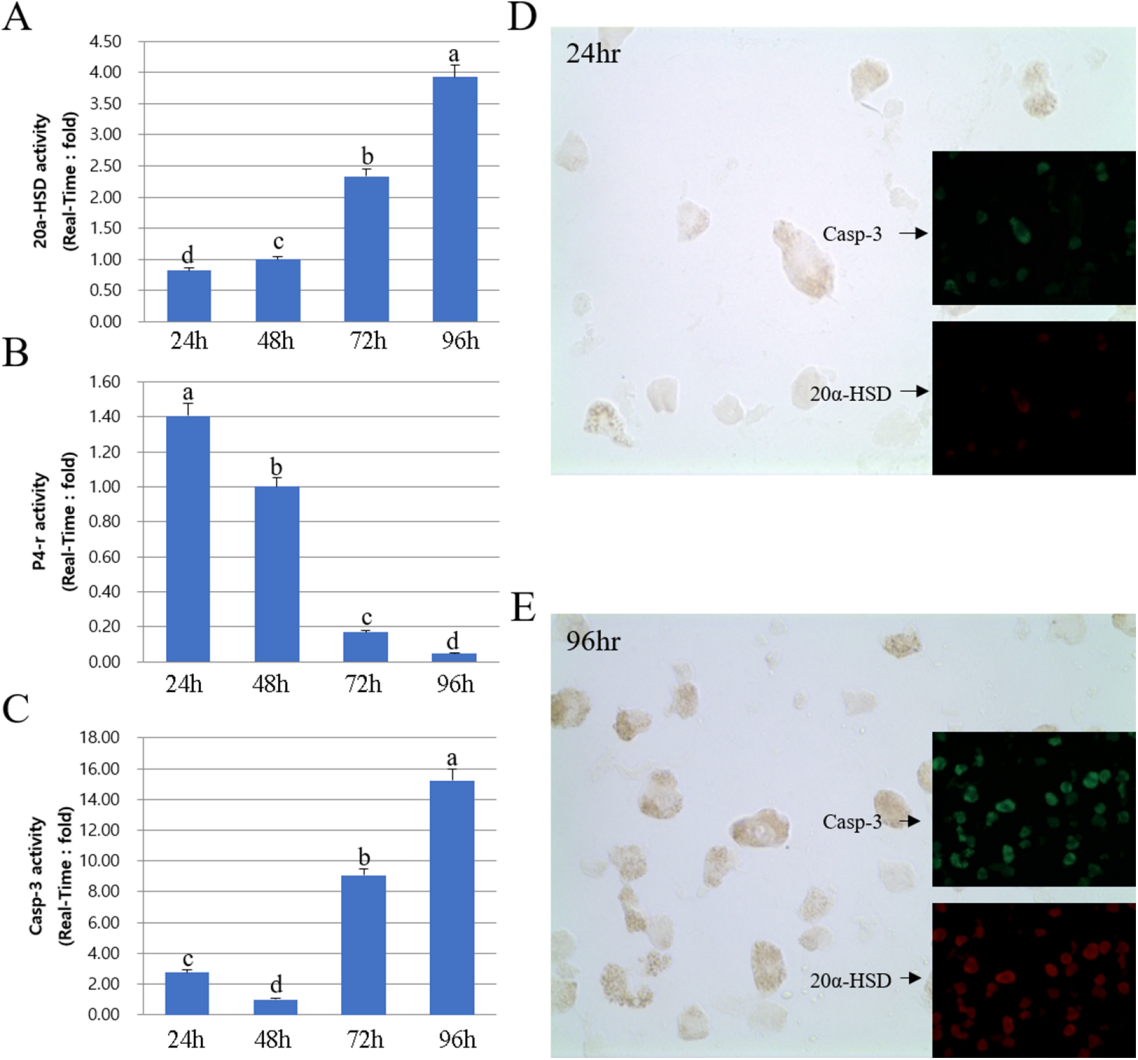
Expression of 20α-HSD and Casp-3 from 24 to 96 h in cultures. The bars represent the average fold changes from three independent experiments (±SD). (A) 20a-HSD mRNA level. (B) P4-r mRNA level. (C) Casp-3 mRNA level. (D) Immuno-fluorescence in cells 24 h after cell culture. (E) Immuno-fluorescence in cells 96 h after cell culture.^a,b,c,d^ Different letters within the same column represent a significant difference (*p* < 0.05).

Next, we compared the protein and mRNA expression levels of 20a-HSD, P4-r, and Casp-3 genes. Overall, the expression profiles of 20α-HSD and Casp-3 were in agreement with their corresponding MMP-2 expression levels. Furthermore, 20α-HSD and Casp-3 protein expression increased from 24 to 96 h. Taken together, the expression of MMPs and TIMPs was associated with that of apoptosis-related genes (20α-HSD and Casp-3) and increased from 24 to 96 h in cultured luteal cells (Fig. 6).

## Discussion

Here we demonstrate the differential expression of MMP-2 and MMP-9 genes during the development and degradation of bovine CL. In particular, the activation of MMP-2 enzyme increased at luteolysis, whereas the expression of MMP-9 was higher during the development of luteal cells.

Regression of CL is one of the most important mechanisms for the return to estrus after parturition and begins with the decrease in the blood level of CL, polyphagia, and PGF2α (McCracken, Custer & Lamsa, 1999). The structural changes in CL include cellular proliferation (Jablonka-Shariff et al., 1993), remodeling of the luteum matrix (Luck & Zhao, 1993), and angiogenesis (Zheng, Redmer & Reynolds, 1993). Although little is known about the factors that coordinate these processes in CL, the biological mechanism underlying these changes has essential roles (Woessner, 1991, 2002). The degradation systems of luteal cells need to be detached from the luteum tissue into the lymphatic vessels. The matrix of CL is composed of collagen, the main component of ECM, and collagen type I is a predominant luteal collagen (Irving-Rodgers et al., 2006). MMPs secreted from cells, such as MMP-14, anchor to the plasma membrane and have the ability to cleave collagen types I, II, and III (Klein & Bischoff, 2011).

For the degradation of luteum tissue, 20α-HSD is expressed in luteal cells of cattle. The gene expression is increased with luteolysis (Kim et al., 2014a). The abrupt increase in the expression of 20α-HSD gene at the end of the pregnancy may be associated, in part, with the concentration of PGF2α, which increases at the end of the pregnancy (Kim et al., 2014b).

The development and degradation of CL plays an important role during pregnancy. Our results suggest that the expression of survival-associated genes is important for the successful development and degradation mechanism in CL cycle. In particular, the expression of LH receptor was induced during CL stages than in CH stages. The expression of FSH receptor was mostly high during CL2 and CL1 stages and the level of mTOR protein was higher during the development of CL. On the other hand, the level of pregnancy-associated PAPP-a mRNA was higher during the last stage of CL. Therefore, our results demonstrate the different expression of genes associated with cell death and survival after the decrease in mTOR, the survival signal marker in the target cells (Levine & Klionsky, 2004).

The activity of MMP-2 gelatinase was higher during the last CL stage, whereas that of MMP-9 was higher during the early stage of luteal phase. Our study indicates that the extracellular composition of CL changes throughout the luteal phase (Parry, Willcox & Thorburn, 1980; Luck & Zhao, 1993). MMP-2 is a gelatinase and plays a key role in various tissue remodeling processes, including trophoblast invasion (Werb et al., 1989) and tumor cell motility (Liotta, Steeg & Stetler-Stevenson, 1991). The persistent activation of MMP-2 throughout the last phase of the estrous cycle supports the hypothesis that tissue remodeling continues to occur throughout the degradation process of CL. Our results showed that TIMP-2 protein expression decreased and MMP-9 expression increased from the early luteal cells (Abe, Sakumoto & Okuda, 2015). However, TIMP-3 (MMP-9 inhibitor) was highly expressed in cells cultured for 96 h, whereas MMP-9 enzyme was no longer detectable. This observation may highlight the relative importance of MMP inhibitors at various stages during the development and degradation of luteal cells (Goldberg, Moses & Tsang, 1996). Overall, the expression profiles of apoptosis-associated genes (20α-HSD and Casp-3) were in agreement with their corresponding MMP-2 expression levels from the degraded luteal cells. Taken together, the expression of MMPs and TIMPs was associated with apoptosis-associated genes (20α-HSD and Casp-3) and increased in luteal cells cultured for 96 h. The change in 20α-HSD and MMP level during CL development and degradation observed here was similar to that reported by Kim et al. (2014a). Based on the expression pattern of MMPs and TIMPs, we propose that MMPs play a role in CL degradation, at least in part, through the regulation of the remodeling system in bovine CL tissue.

## Conclusion

We report the luteal cells undergoing continual structural changes activate MMPs during development, resulting in the degradation of CL tissue. In the study results, the activity of MMP-2 was high in the latter corpus luteal (CL2 and CL1), whereas MMP-9 was highly active in the early corpus luteal (CH2 and CH3). Also, RT-PCR results for MMP and TIMP expression patterns in cultured luteal cells were similar to immunofluorescence results. In particular, the expression profiles of 20α-HSD and Casp-3 were in agreement with their corresponding MMP-2 expression levels from the degraded luteal cells. Also, the expression of MMPs was associated with apoptosis-associated genes and increased in luteal cells cultured for 96 h. In this results indicate that MMPs activation patterns can be used as an specific marker in CL remodeling. Based data’s on the activation of MMPs and TIMPs, we propose that MMPs plays a role in the lutein cell remodeling system at least partially by regulating the selective regression in bovine CL.

## Supplemental Information

10.7717/peerj.6344/supp-1 Supplemental Information 1 Raw data exported from the ELISA of corpus luteum applied for data analyses and preparation for the detailed investigation shown in Fig. 1.

10.7717/peerj.6344/supp-2 Supplemental Information 2 Raw data exported from the real-time PCR of corpus luteum applied for data analyses and preparation for the detailed investigation shown in Figs. 2 and 3.

10.7717/peerj.6344/supp-3 Supplemental Information 3 Raw data exported from the real-time PCR applied for data analyses and preparation for the detailed investigation shown in Figs. 4–6 for the time period of 24–96 h.

10.7717/peerj.6344/supp-4 Supplemental Information 4 Raw data exported from the zymography of corpus luteum applied for data analyses and preparation for the detailed investigation shown in Fig. 3.

10.7717/peerj.6344/supp-5 Supplemental Information 5 Raw data exported from the immunohistochemistry of corpus luteum applied for data analyses and preparation for the detailed investigation shown in Fig. 3. Location and expression pattern analysis of MMP protein in corpus luteum stage, raw data for Fig. 3A (Compression file #1).

10.7717/peerj.6344/supp-6 Supplemental Information 6 Raw data exported from the immunohistochemistry of corpus luteum applied for data analyses and preparation for the detailed investigation shown in Fig. 3. Location and expression pattern analysis of MMP protein in corpus luteum stage, raw data for Fig. 3A (Compression file #2).

10.7717/peerj.6344/supp-7 Supplemental Information 7 Raw data exported from the immunohistochemistry of corpus luteum applied for data analyses and preparation for the detailed investigation shown in Fig. 3. Location and expression pattern analysis of MMP protein in corpus luteum stage, raw data for Fig. 3A (Compression file #3).

10.7717/peerj.6344/supp-8 Supplemental Information 8 Raw data exported from the immunohistochemistry of corpus luteum applied for data analyses and preparation for the detailed investigation shown in Fig. 3. Location and expression pattern analysis of MMP protein in corpus luteum stage, raw data for Fig. 3A (Compression file #4).

10.7717/peerj.6344/supp-9 Supplemental Information 9 Raw data exported from the immunofluorescence of corpus luteum cell applied for data analyses and preparation for the detailed investigation shown in Fig. 4 for the time period of 24–96 h.

10.7717/peerj.6344/supp-10 Supplemental Information 10 Raw data exported from the immunofluorescence of corpus luteum cell applied for data analyses and preparation for the detailed investigation shown in Fig. 5 for the time period of 24–96 h.

10.7717/peerj.6344/supp-11 Supplemental Information 11 Raw data exported from the immunofluorescence of corpus luteum cell applied for data analyses and preparation for the detailed investigation shown in Fig. 6 for the time period of 24 and 96 h.

## Additional Information and Declarations

### Data Availability

The following information was supplied regarding data availability: The raw data are available in the Supplemental Files.
