# Supplementary figures and images for: Expression of matrix metalloproteinases to induce the expression of genes associated with apoptosis during corpus luteum development in bovine
Source: PeerJ. 2019 Jan 30;7:e6344. doi: 10.7717/peerj.6344 (PMC6361312; doi:10.7717/peerj.6344)

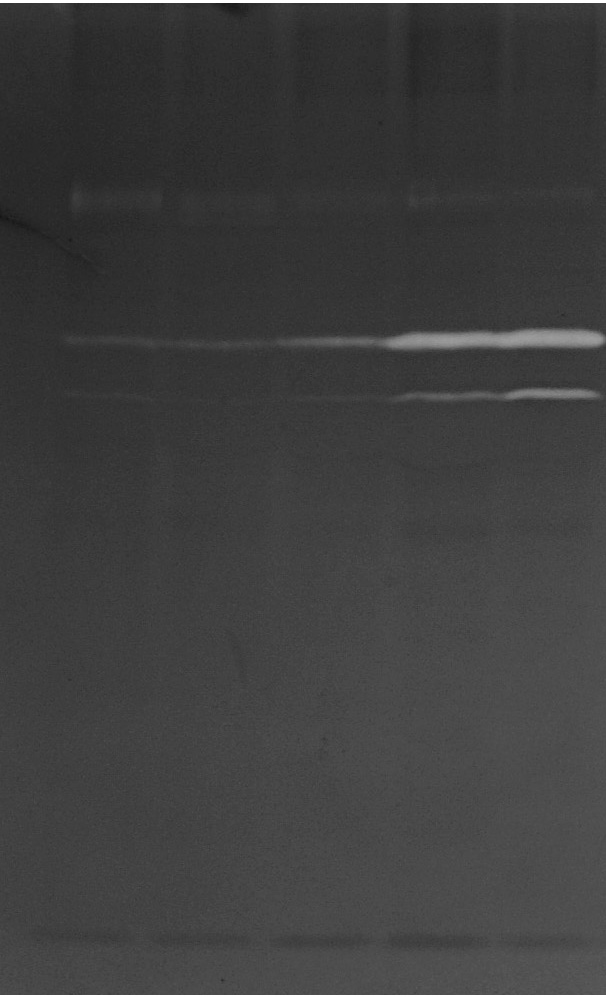

Supplement: Supplemental Information 4 [file peerj-07-6344-s004.zip › peerj-28581-CL-MMP-2-zymo.jpg]

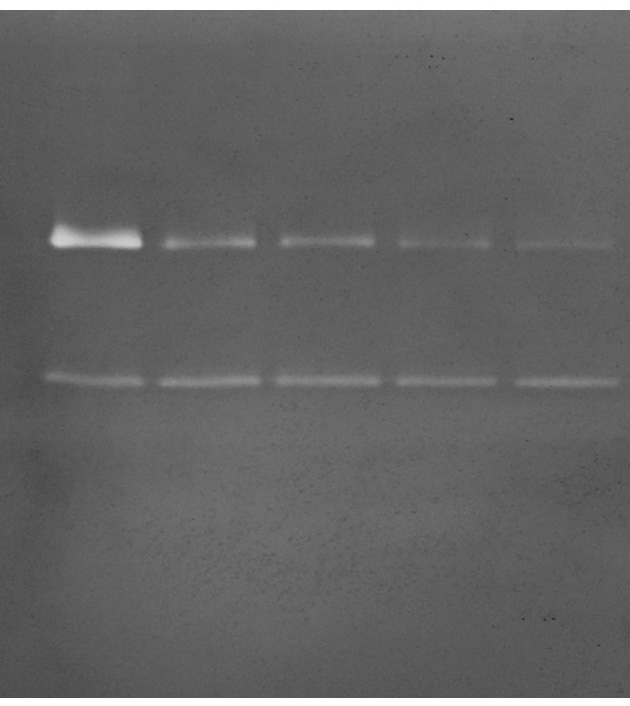

Supplement: Supplemental Information 4 [file peerj-07-6344-s004.zip › peerj-28581-CL-MMP-9-zymo.jpg]

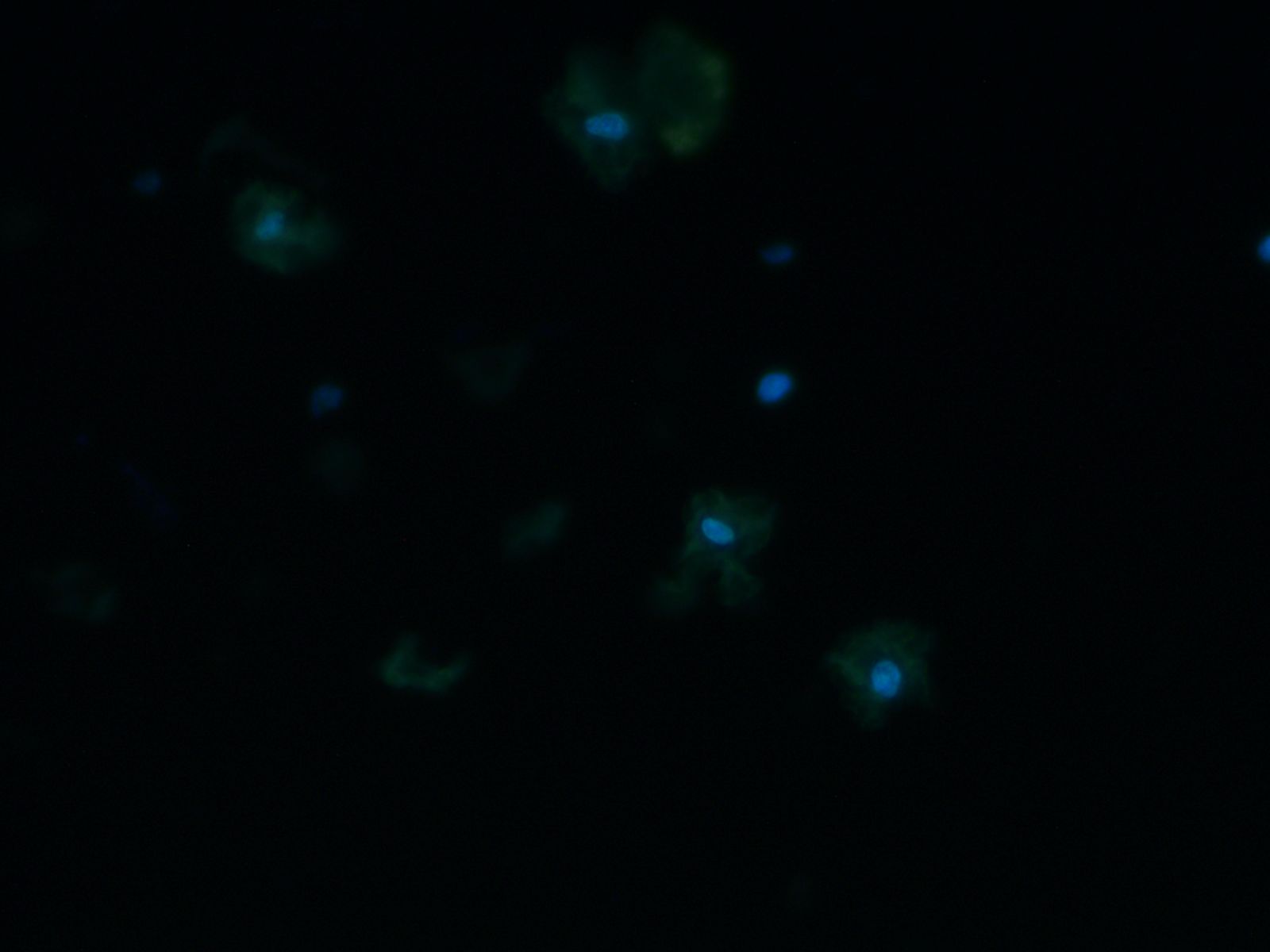

Supplement: Supplemental Information 9 [file peerj-07-6344-s009.zip › 24hMMP-2,9x20(H).jpg]

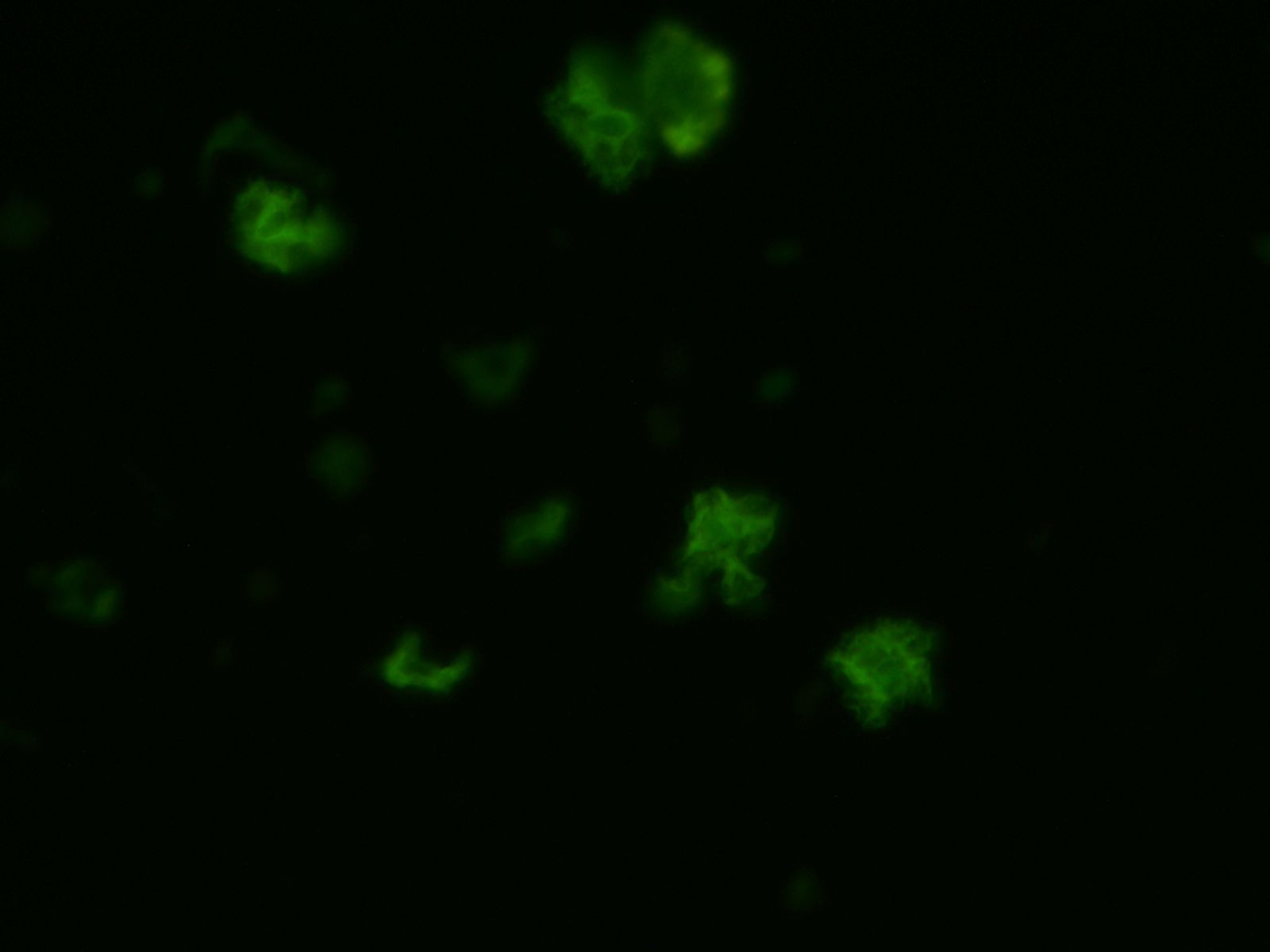

Supplement: Supplemental Information 9 [file peerj-07-6344-s009.zip › 24hMMP-2x20.jpg]

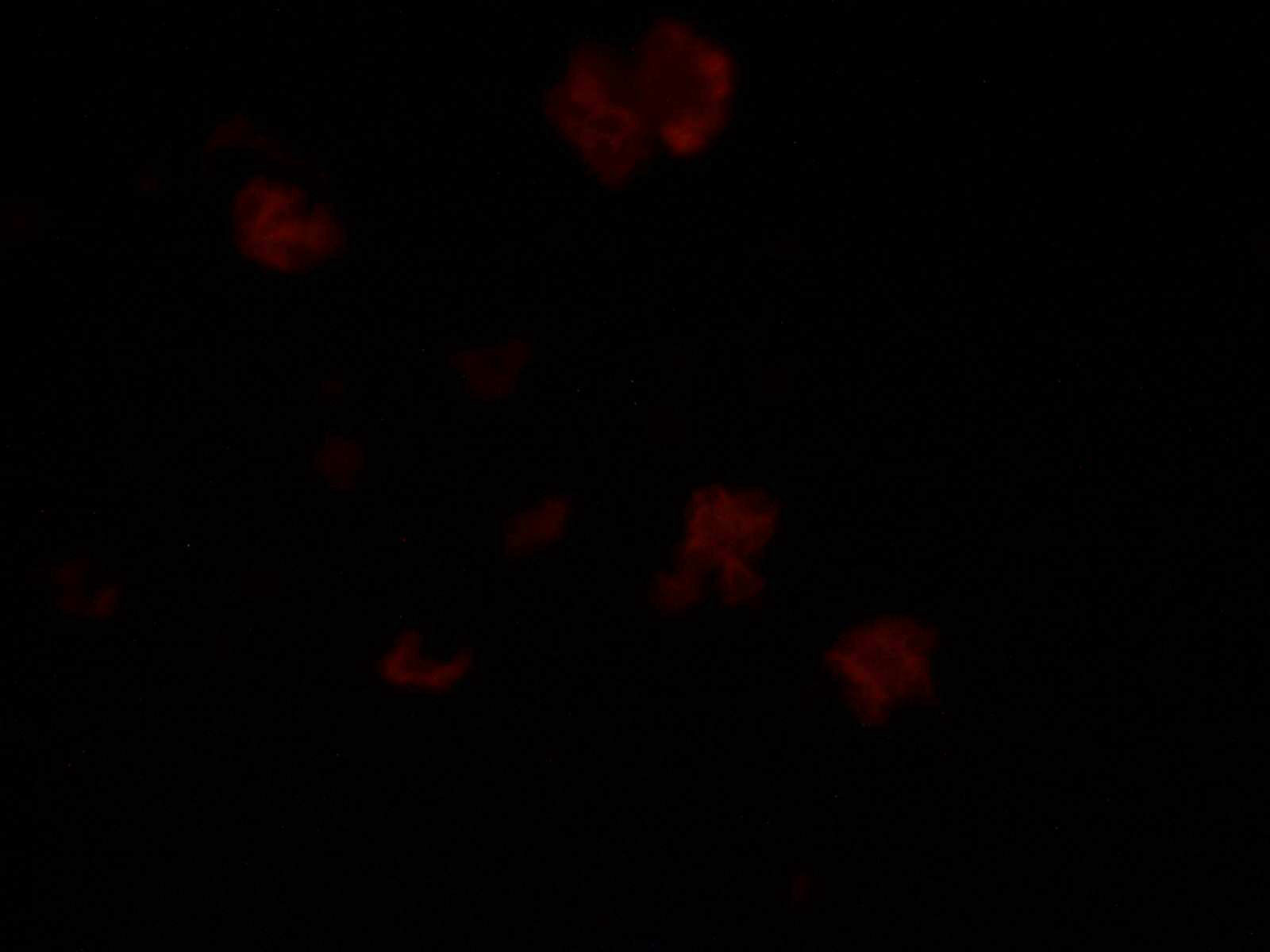

Supplement: Supplemental Information 9 [file peerj-07-6344-s009.zip › 24hMMP-9x20.jpg]

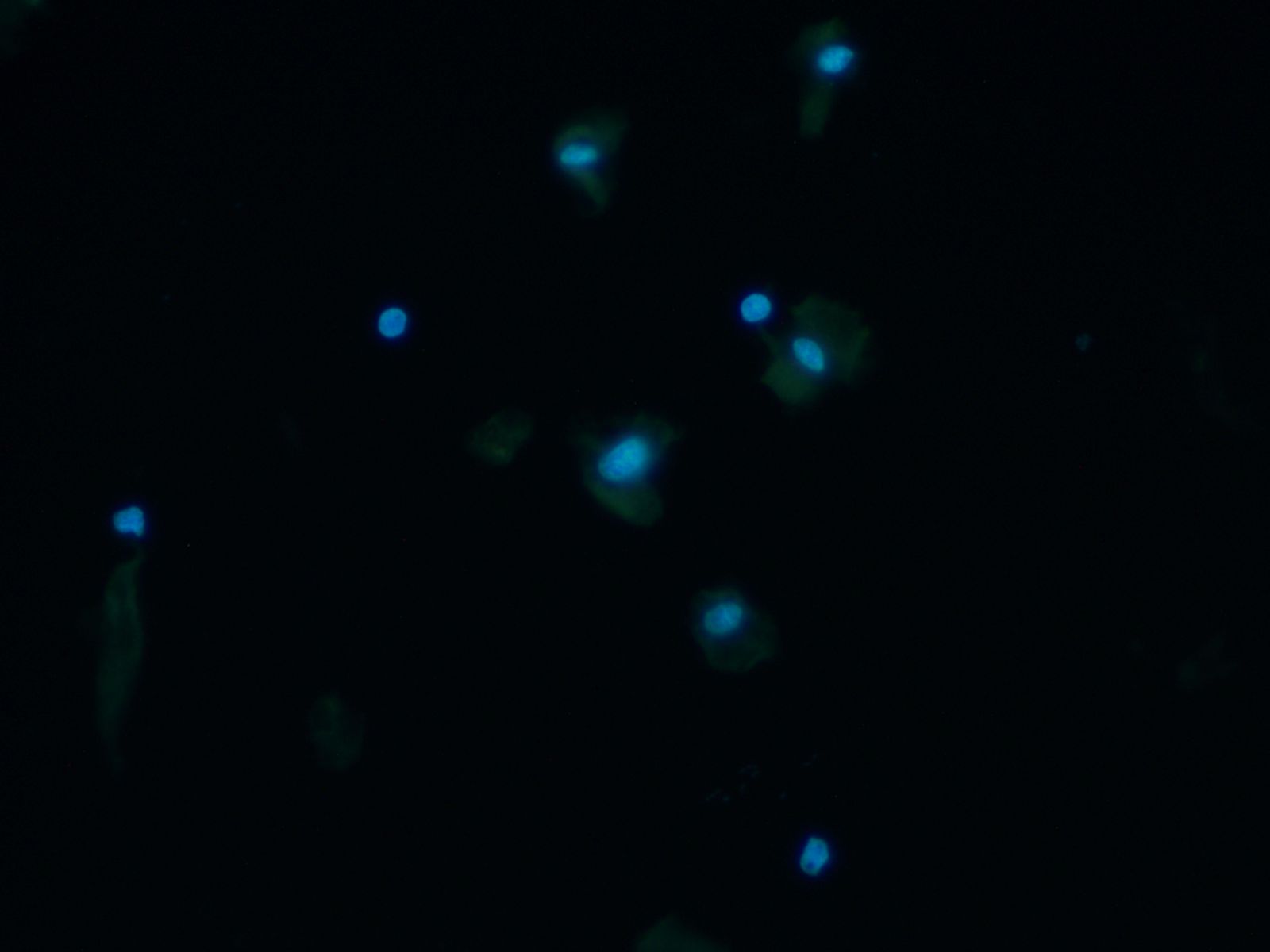

Supplement: Supplemental Information 9 [file peerj-07-6344-s009.zip › 48hMMP-2,9x20(H).jpg]

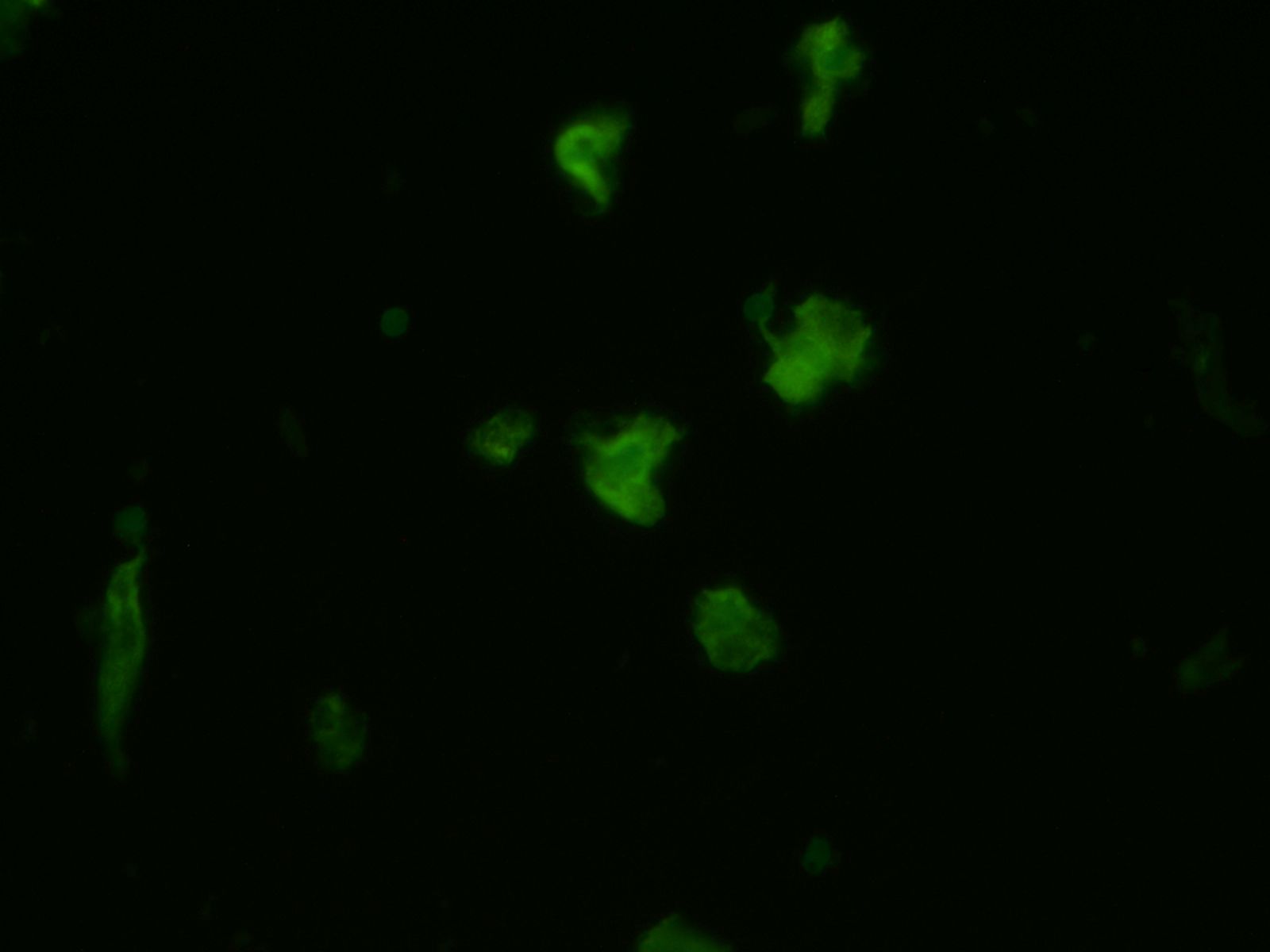

Supplement: Supplemental Information 9 [file peerj-07-6344-s009.zip › 48hMMP-2x20.jpg]

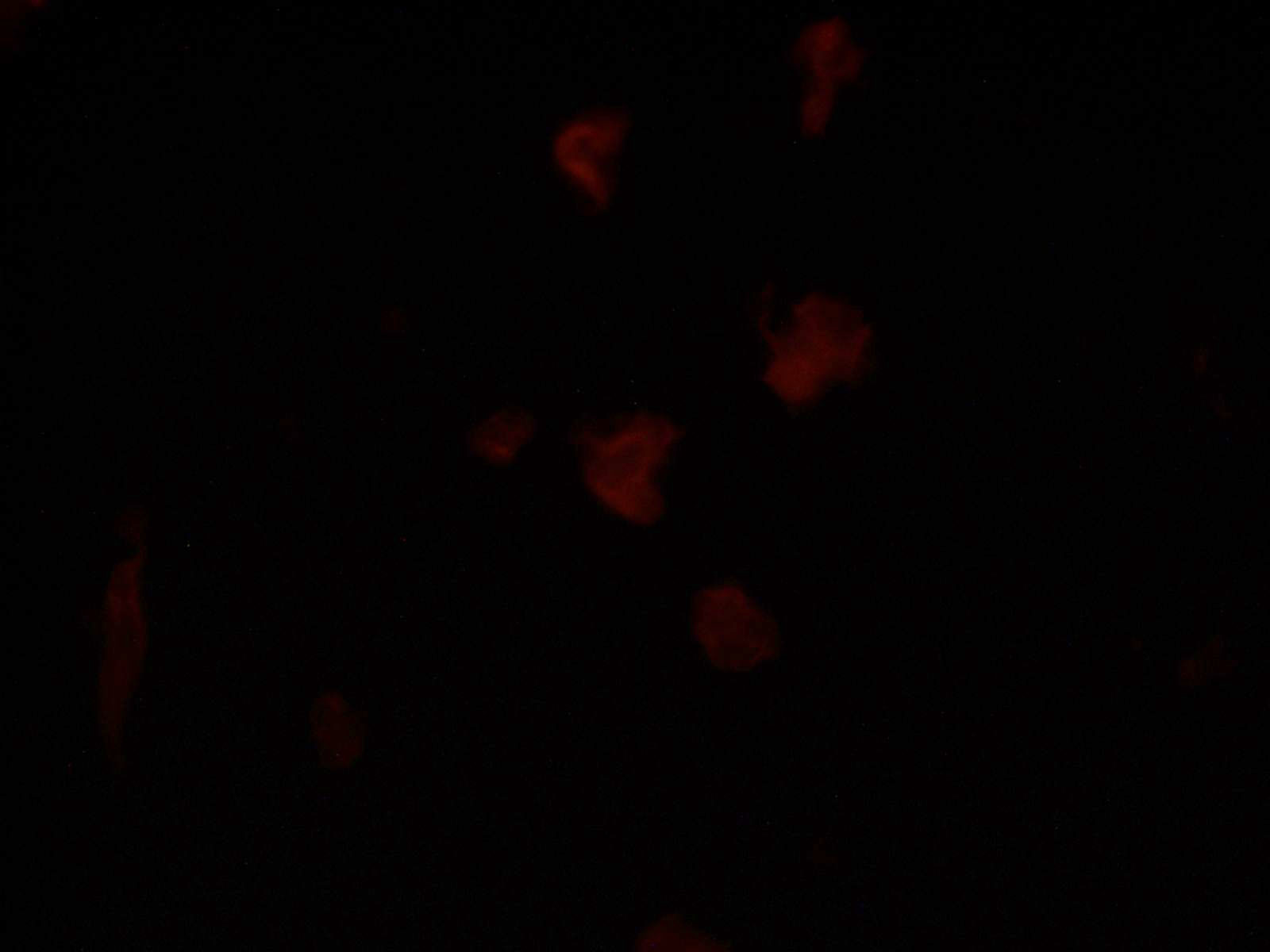

Supplement: Supplemental Information 9 [file peerj-07-6344-s009.zip › 48hMMP-9x20.jpg]

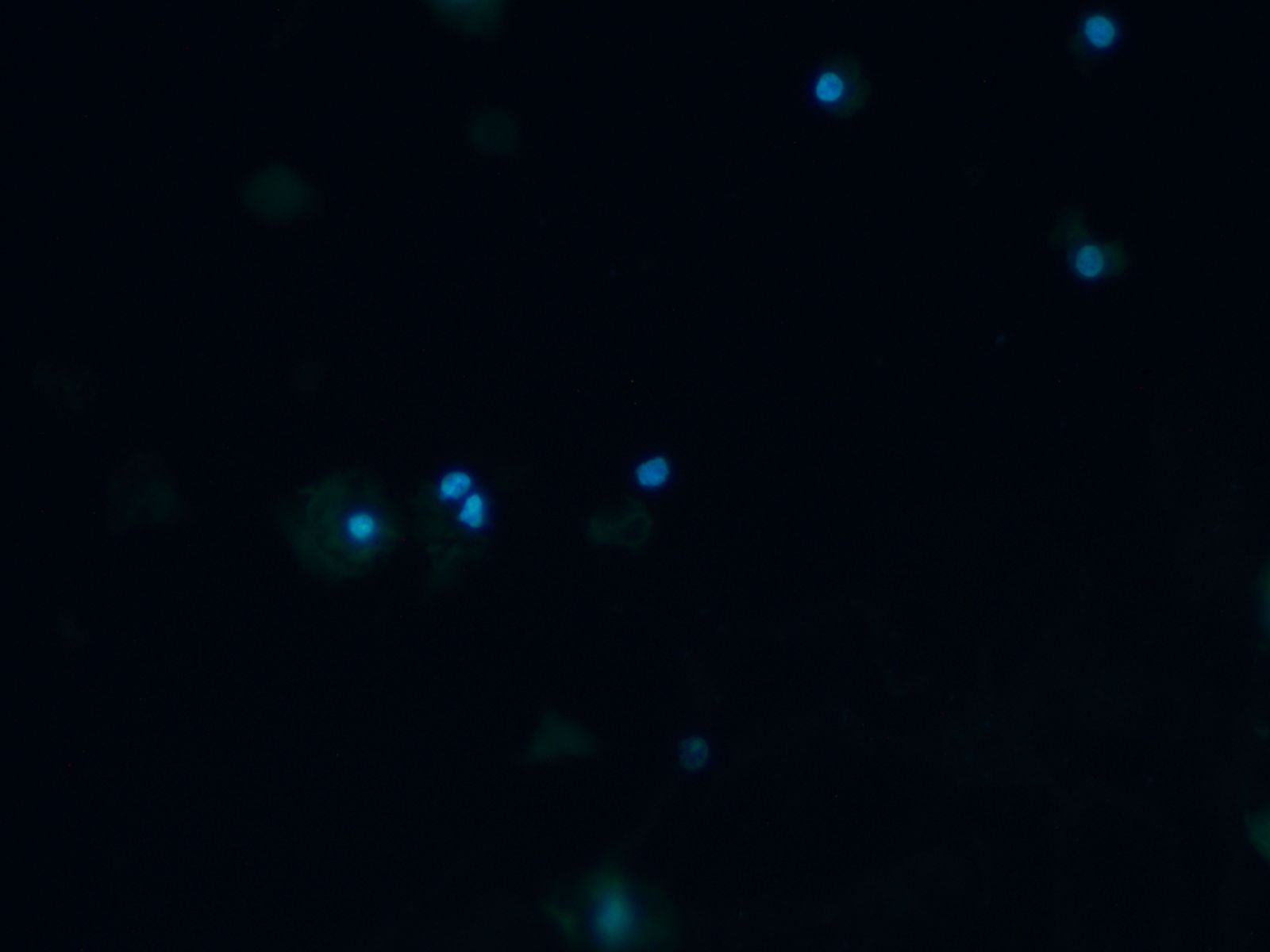

Supplement: Supplemental Information 9 [file peerj-07-6344-s009.zip › 72hMMP-2,9x20(H).jpg]

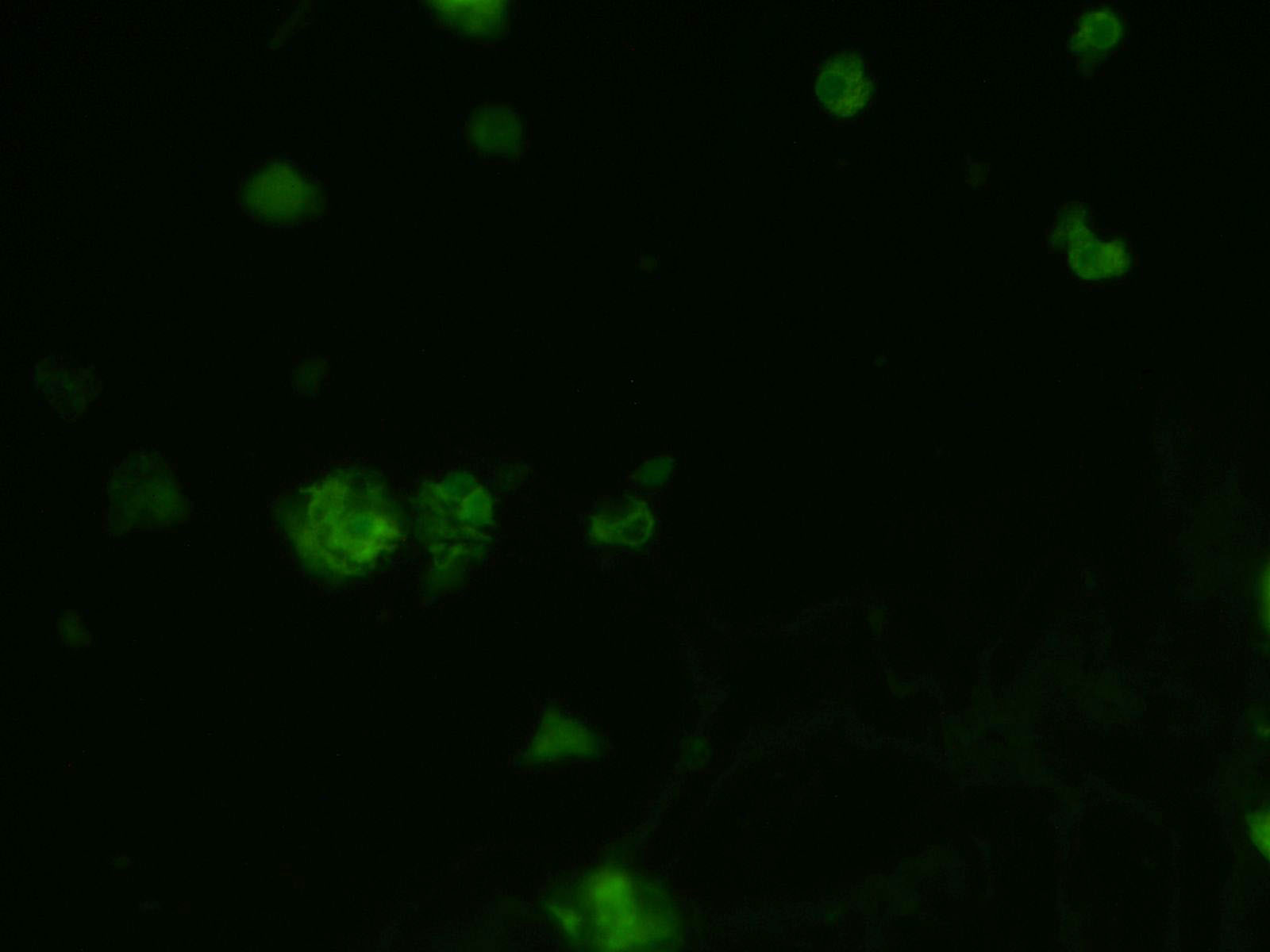

Supplement: Supplemental Information 9 [file peerj-07-6344-s009.zip › 72hMMP-2x20.jpg]

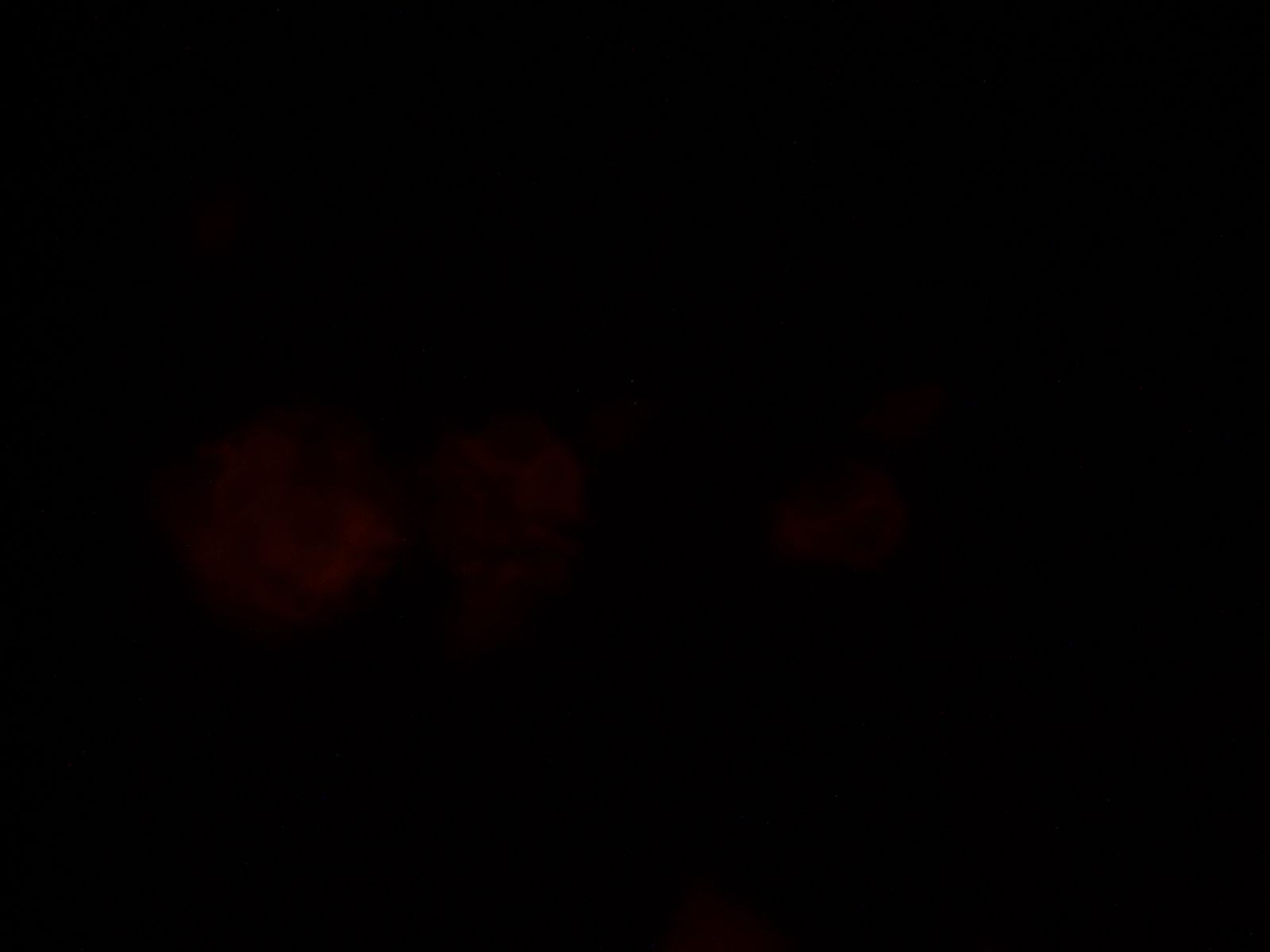

Supplement: Supplemental Information 9 [file peerj-07-6344-s009.zip › 72hMMP-9x40.jpg]

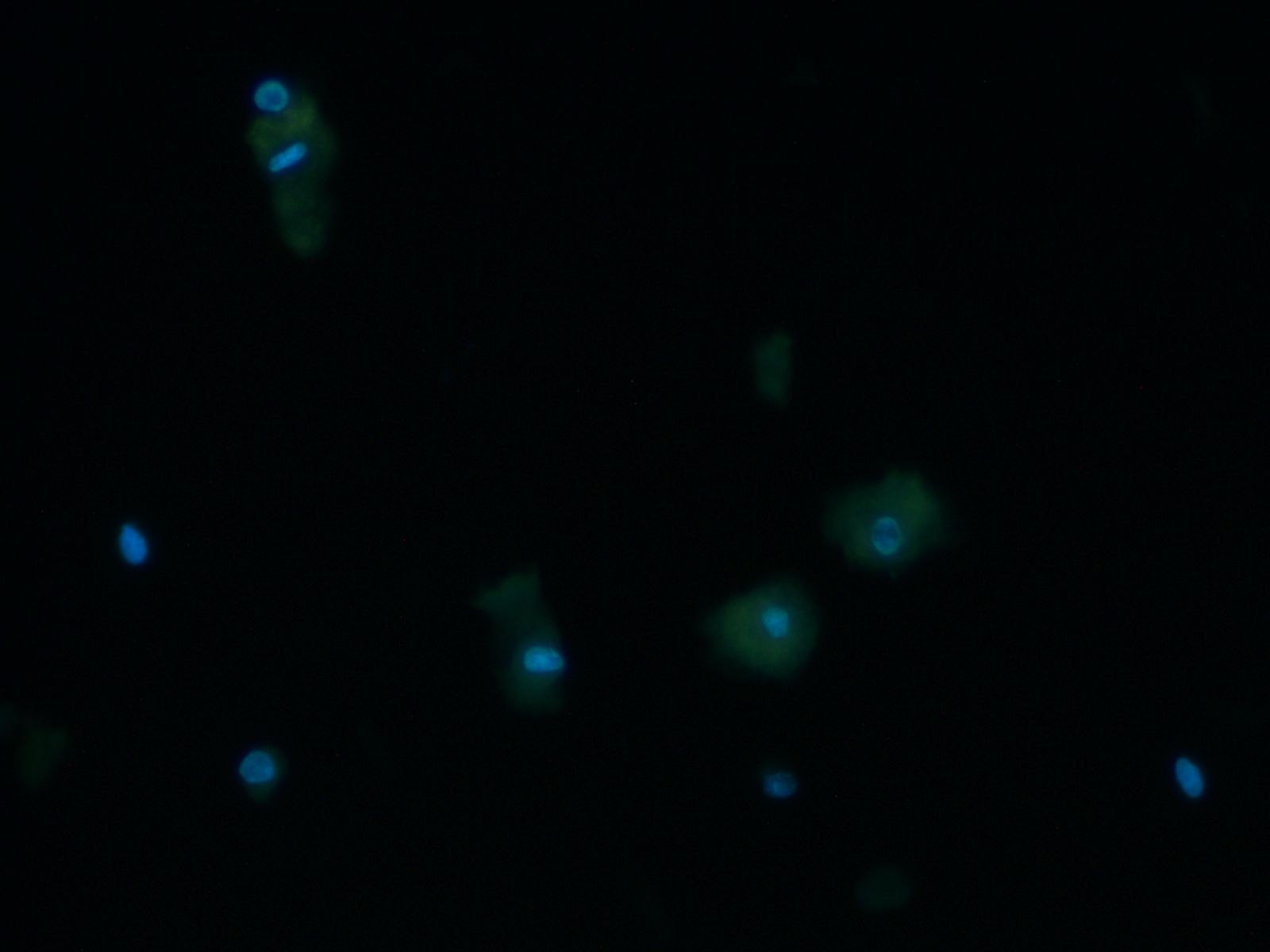

Supplement: Supplemental Information 9 [file peerj-07-6344-s009.zip › 96hMMP-2,9x20(H).jpg]

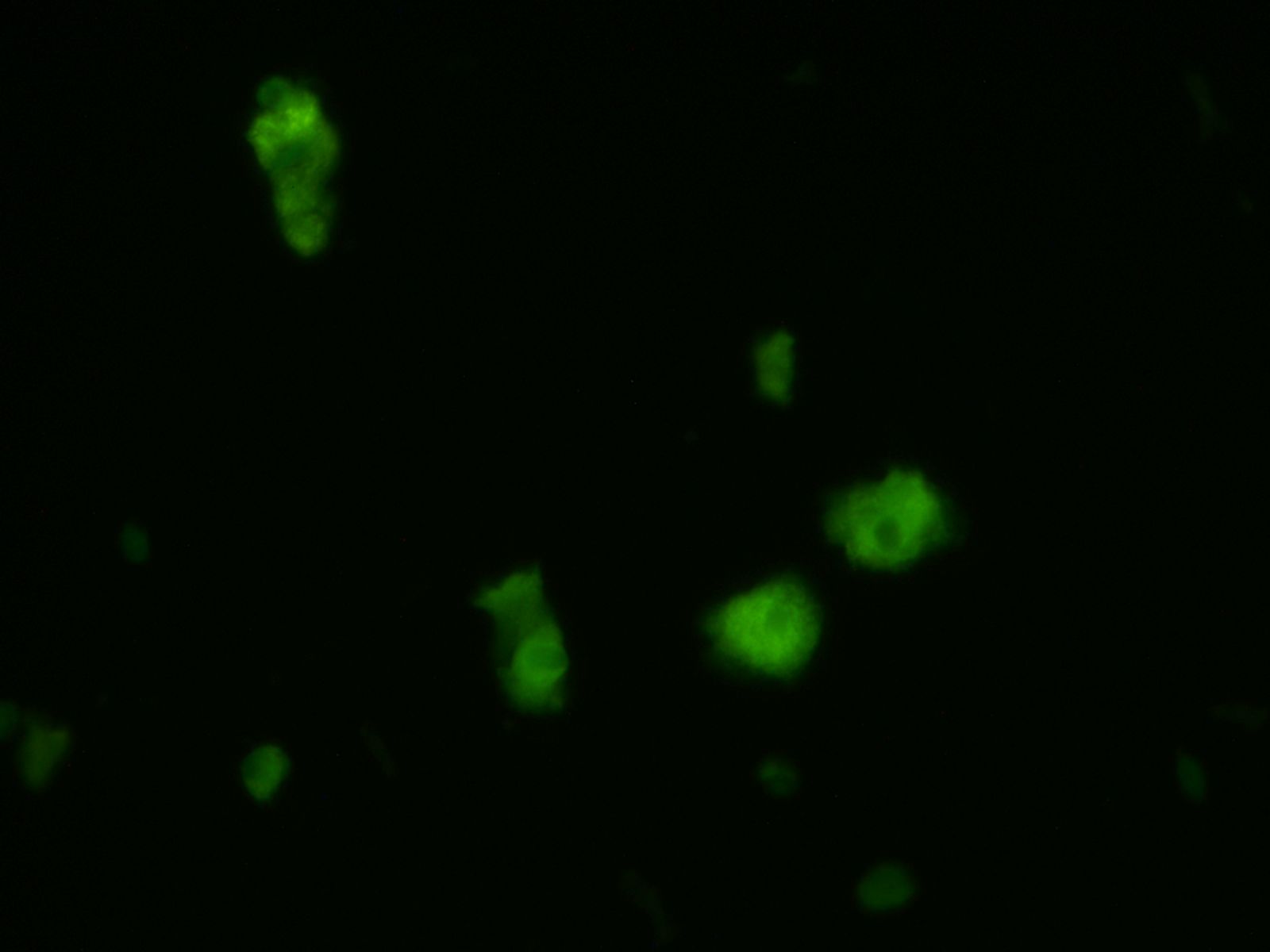

Supplement: Supplemental Information 9 [file peerj-07-6344-s009.zip › 96hMMP-2x20.jpg]

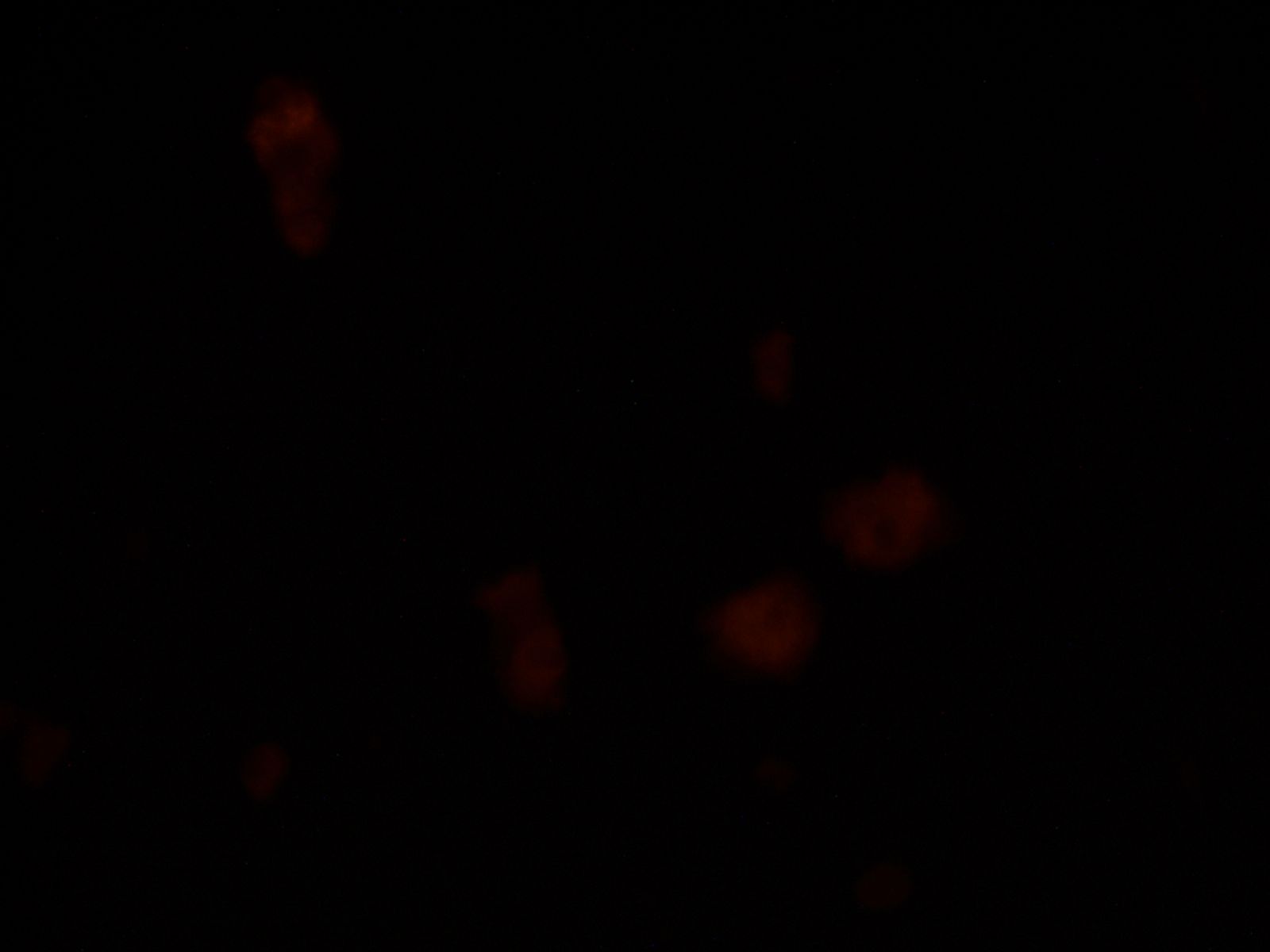

Supplement: Supplemental Information 9 [file peerj-07-6344-s009.zip › 96hMMP-9x20.jpg]

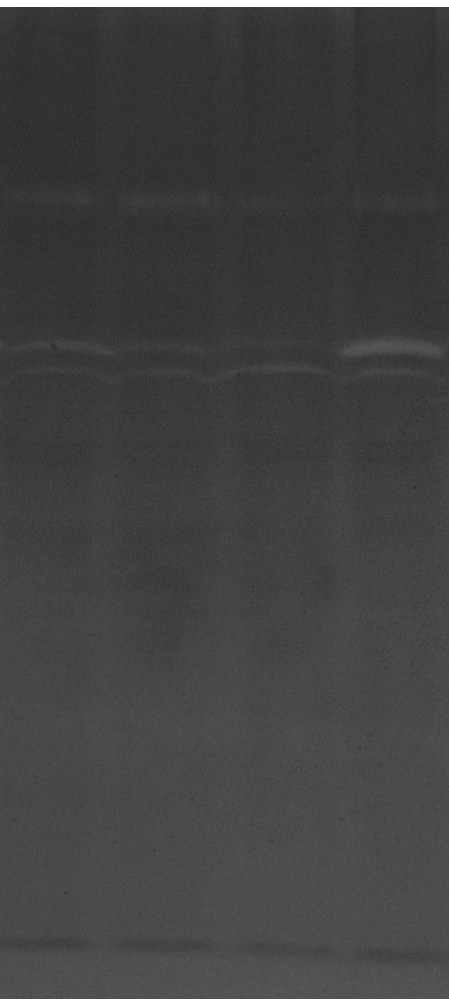

Supplement: Supplemental Information 9 [file peerj-07-6344-s009.zip › cell-MMP-2-zymo.jpg]

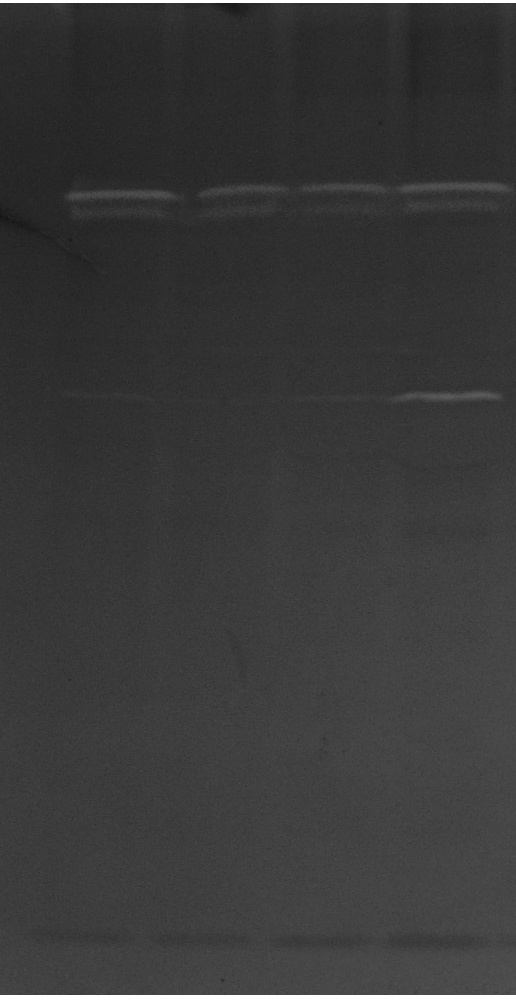

Supplement: Supplemental Information 9 [file peerj-07-6344-s009.zip › cell-MMP-9-zymo.jpg]

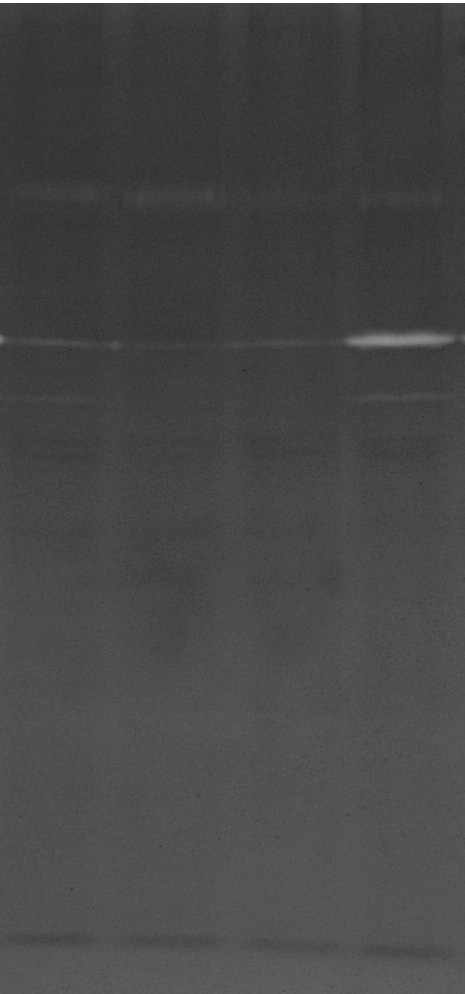

Supplement: Supplemental Information 9 [file peerj-07-6344-s009.zip › media-MMP-2-zymo.jpg]

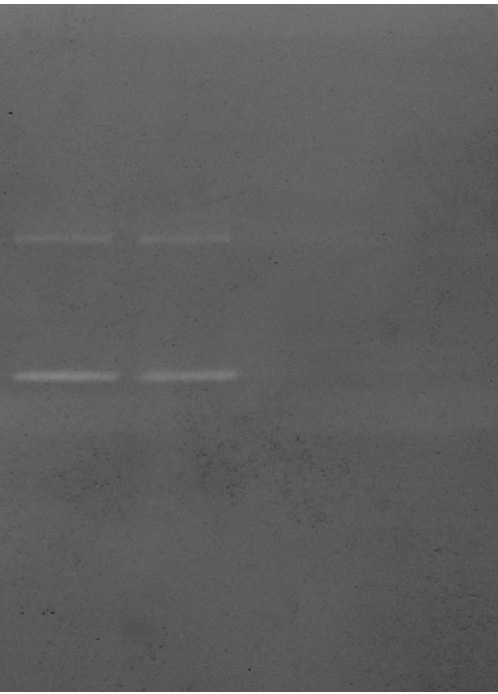

Supplement: Supplemental Information 9 [file peerj-07-6344-s009.zip › media-MMP-9-zymo.jpg]

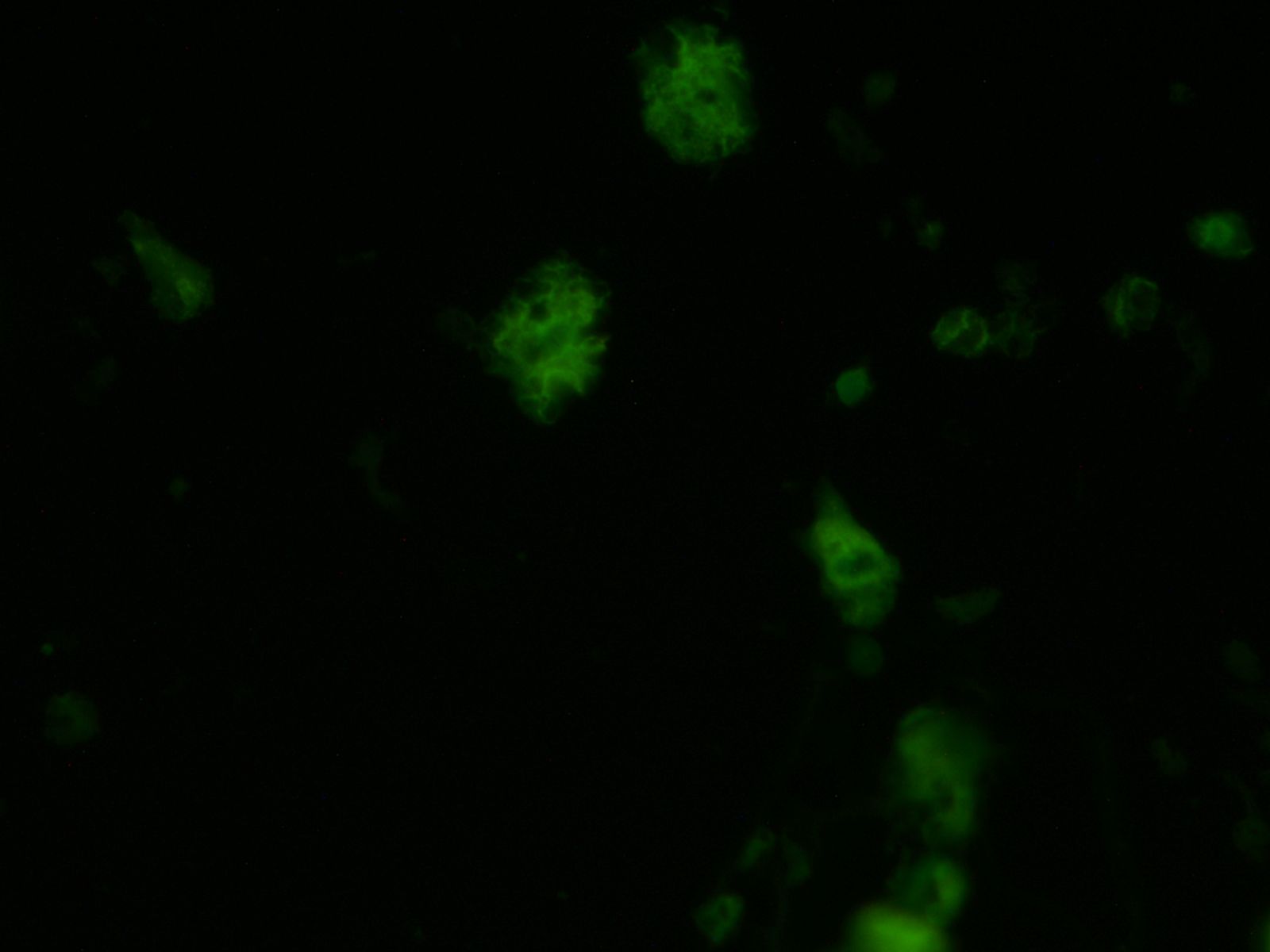

Supplement: Supplemental Information 10 [file peerj-07-6344-s010.zip › 24H Timp-2 20X.jpg]

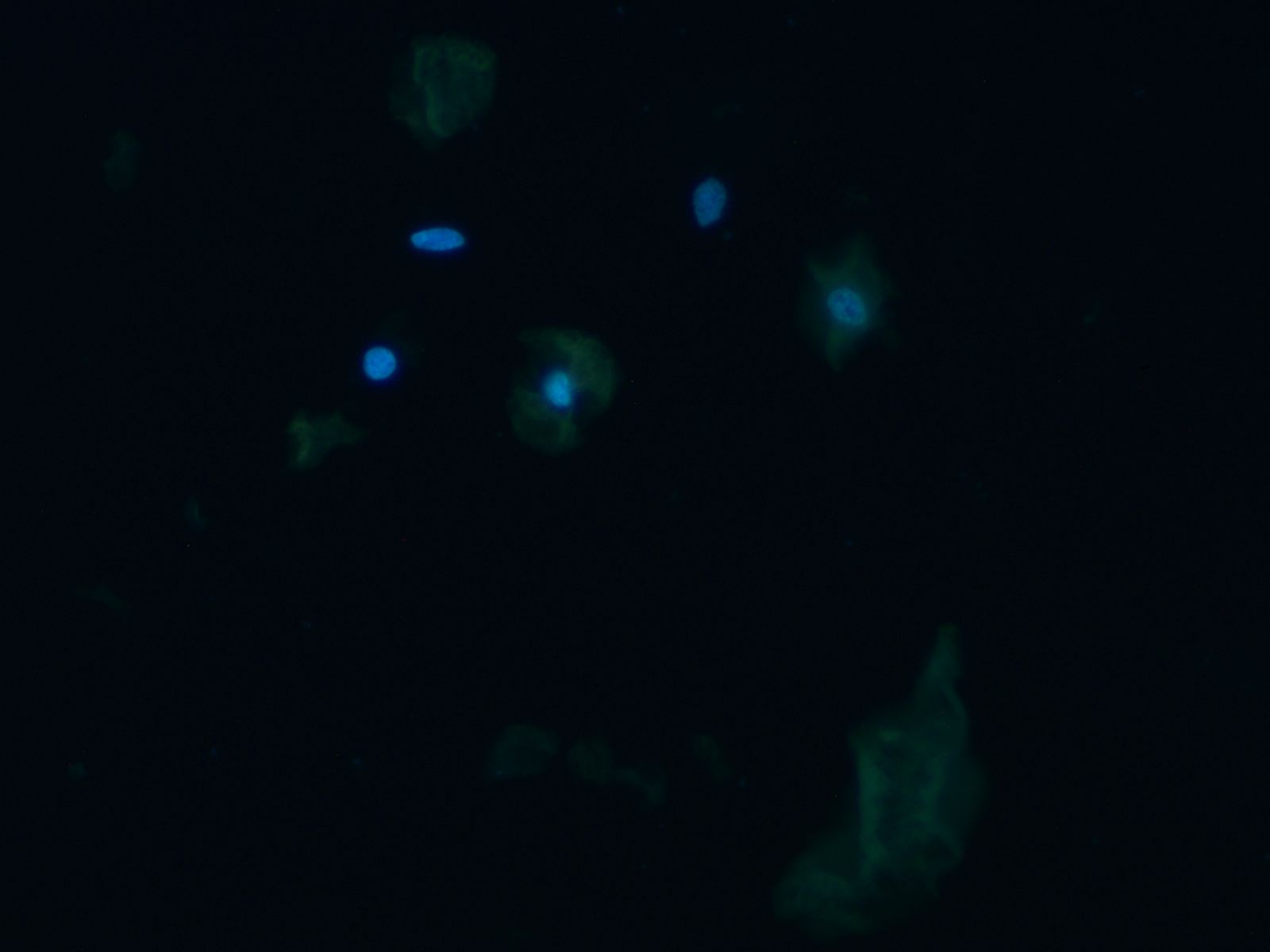

Supplement: Supplemental Information 10 [file peerj-07-6344-s010.zip › 24H Timp-23 20X(H).jpg]

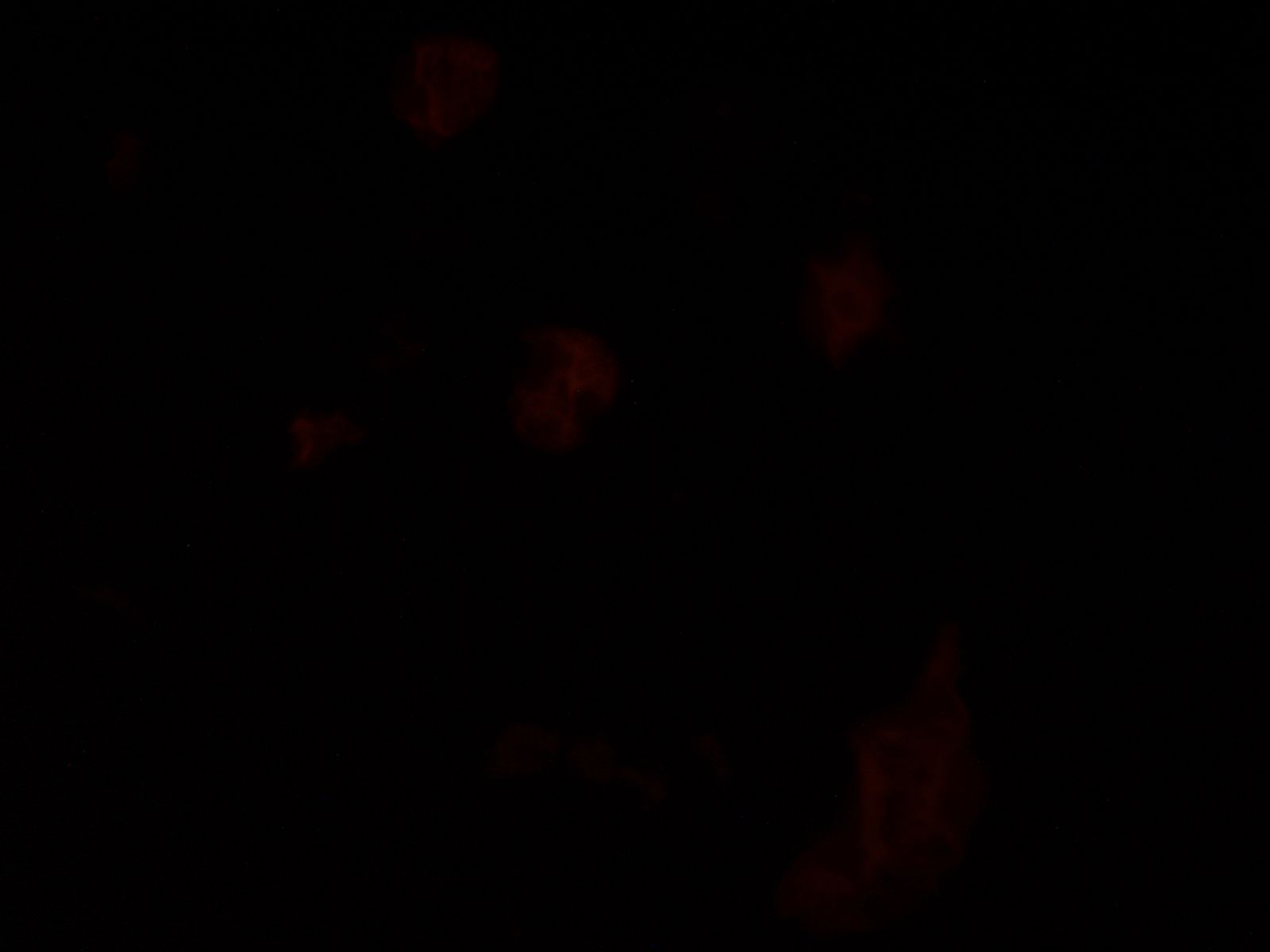

Supplement: Supplemental Information 10 [file peerj-07-6344-s010.zip › 24H Timp-3 20X.jpg]

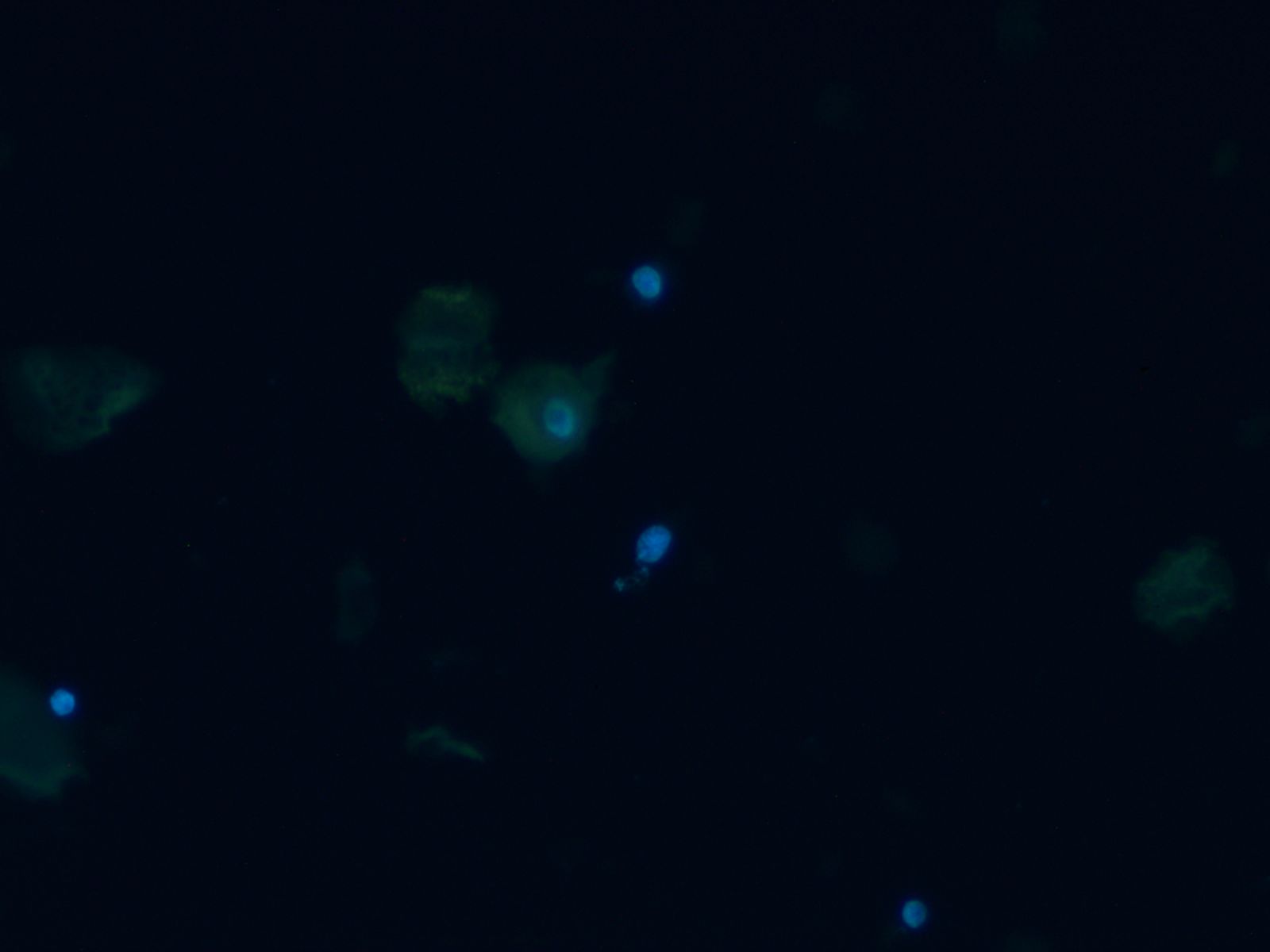

Supplement: Supplemental Information 10 [file peerj-07-6344-s010.zip › 48H TIMP-23 20X(H).jpg]

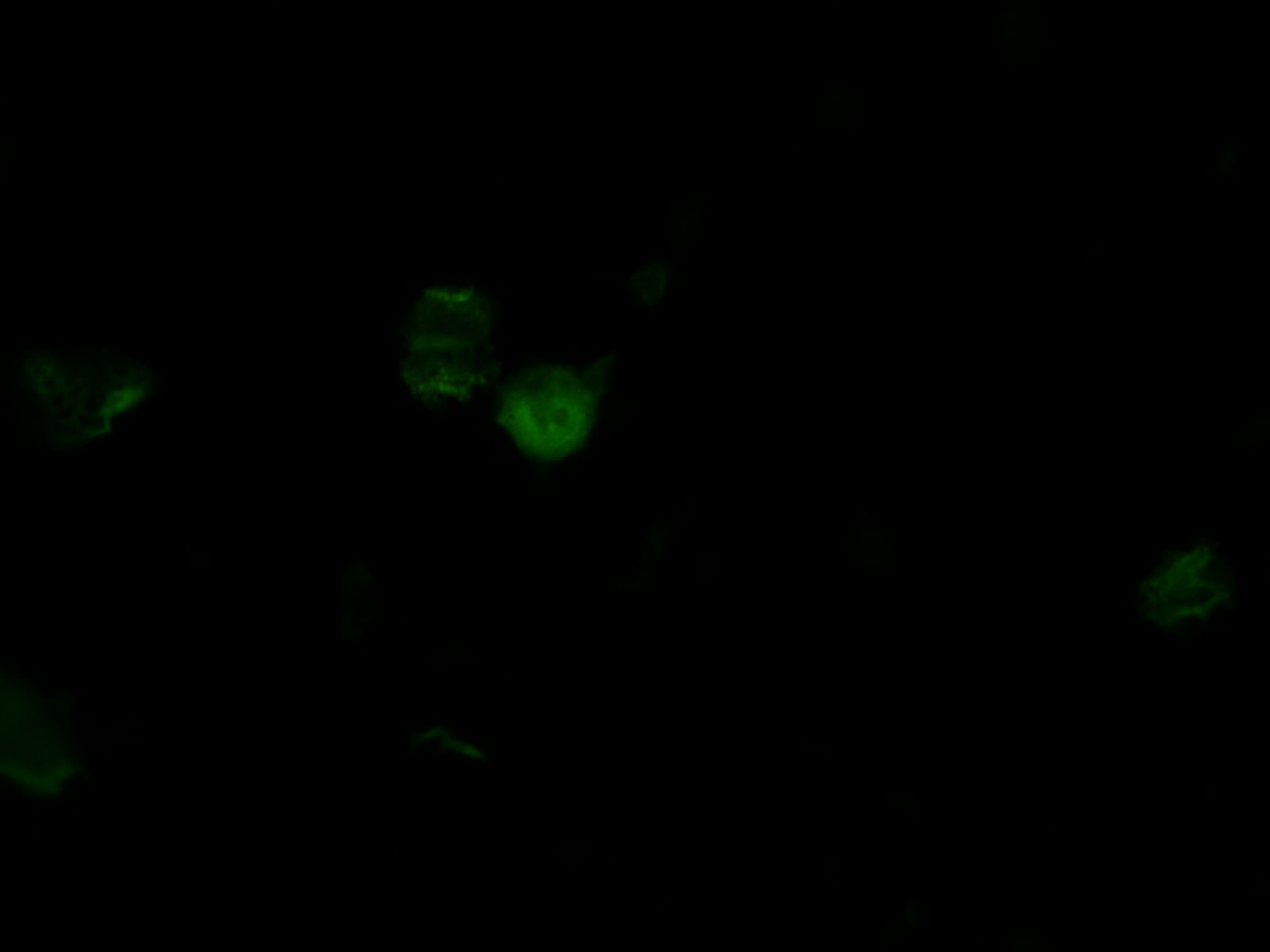

Supplement: Supplemental Information 10 [file peerj-07-6344-s010.zip › 48H Timp-2 20X.jpg]

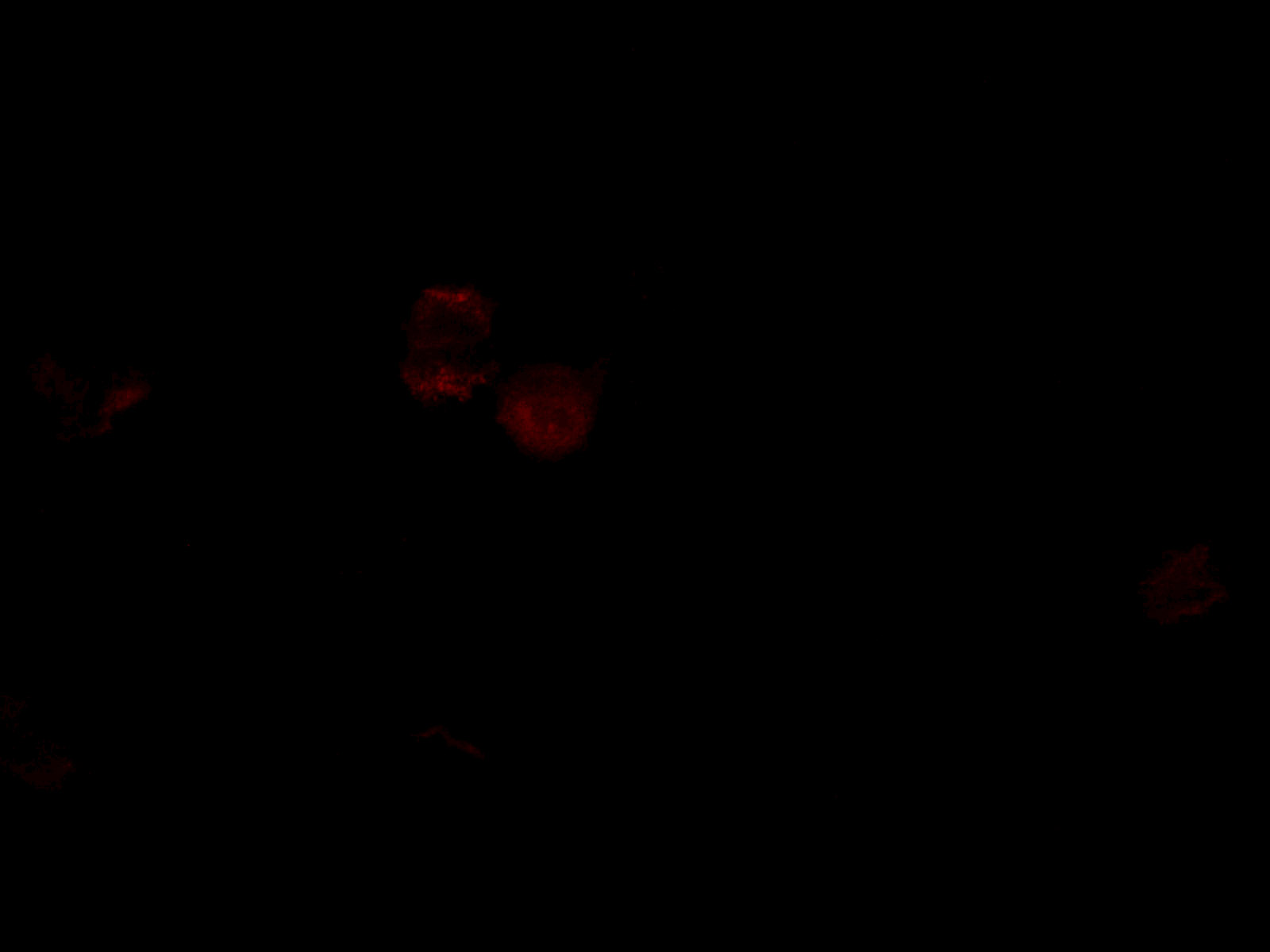

Supplement: Supplemental Information 10 [file peerj-07-6344-s010.zip › 48H Timp-3 20X.jpg]

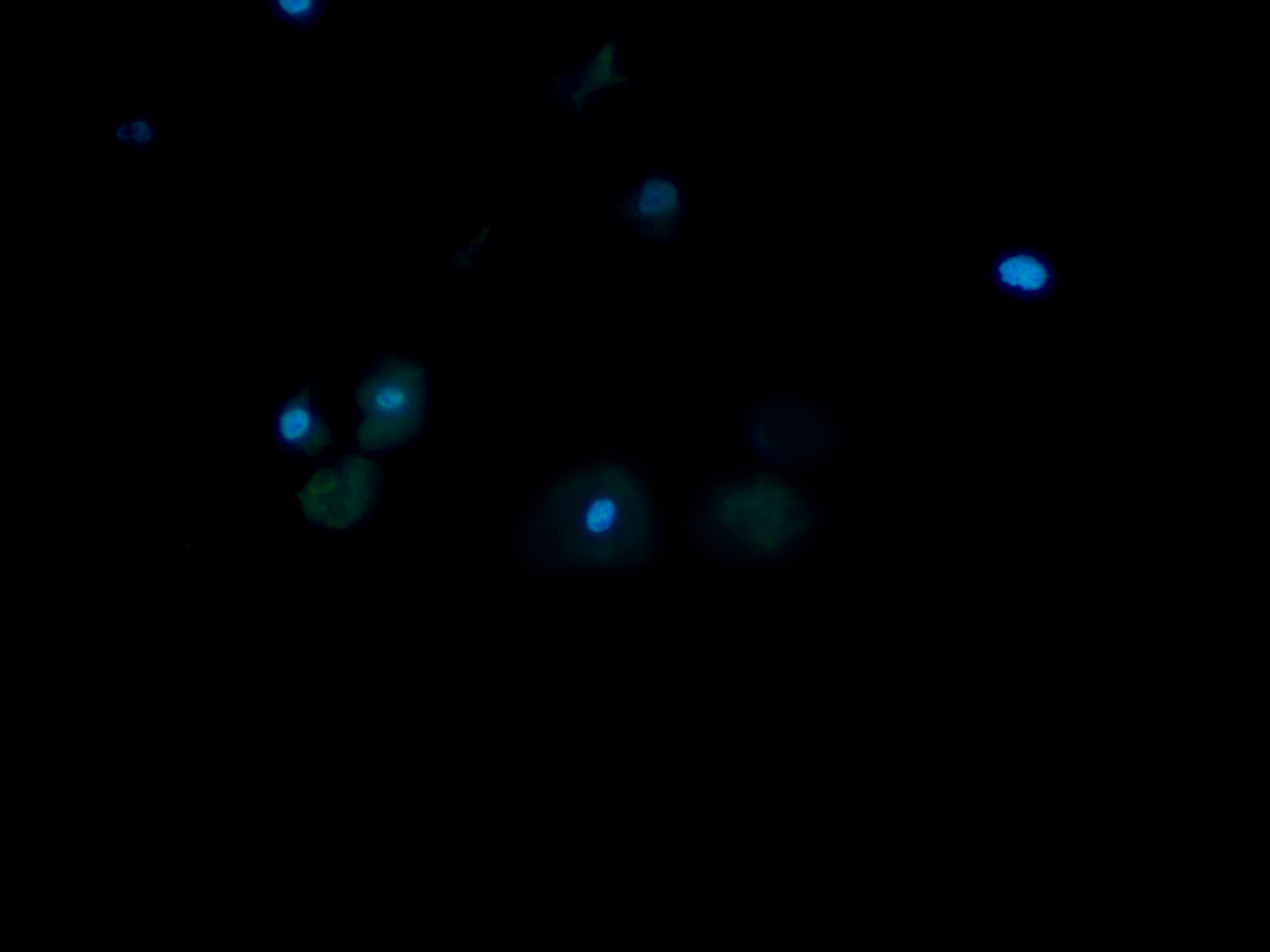

Supplement: Supplemental Information 10 [file peerj-07-6344-s010.zip › 72H TIMP-23 20X(H).jpg]

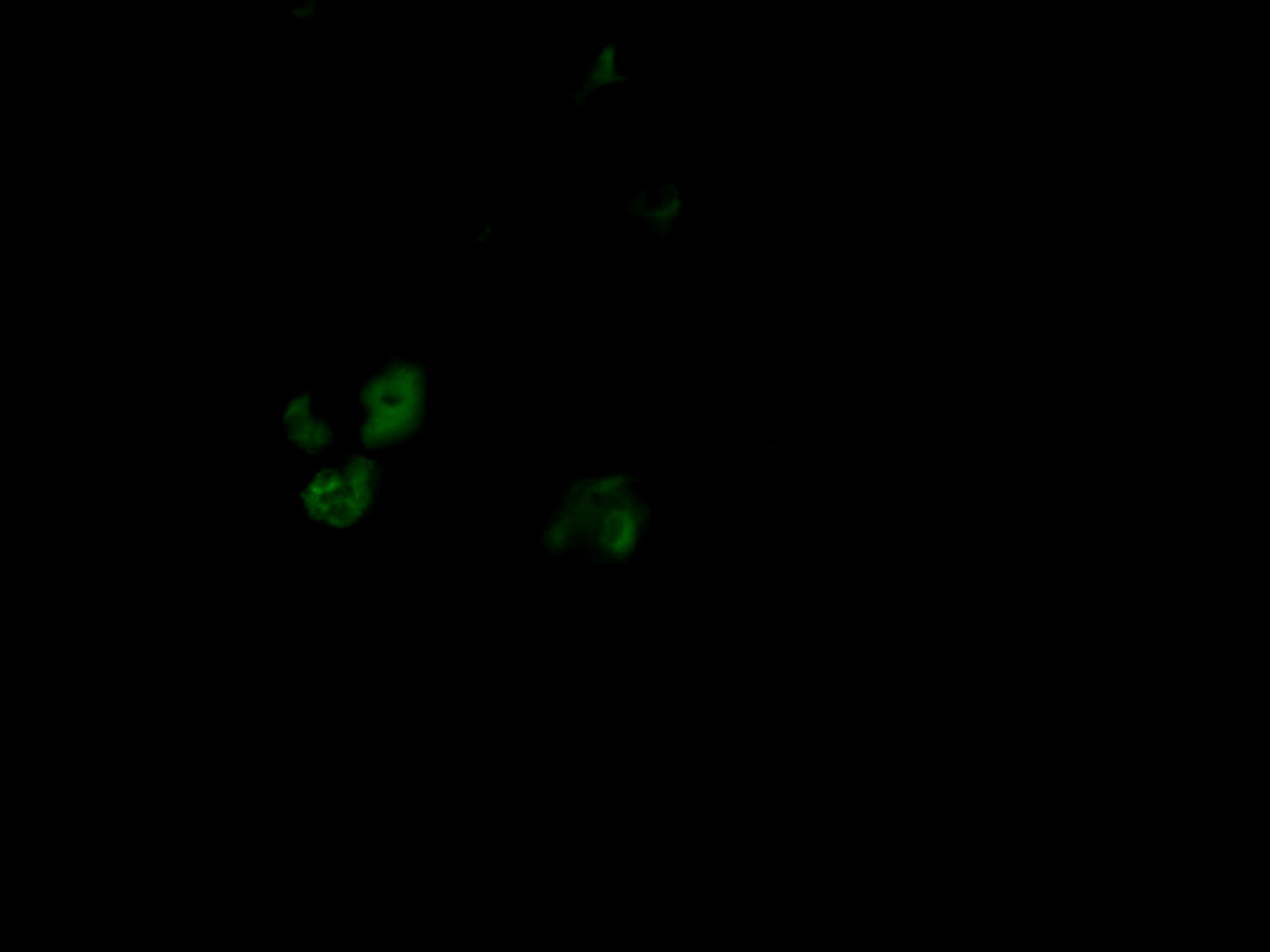

Supplement: Supplemental Information 10 [file peerj-07-6344-s010.zip › 72H Timp-2 20X.jpg]

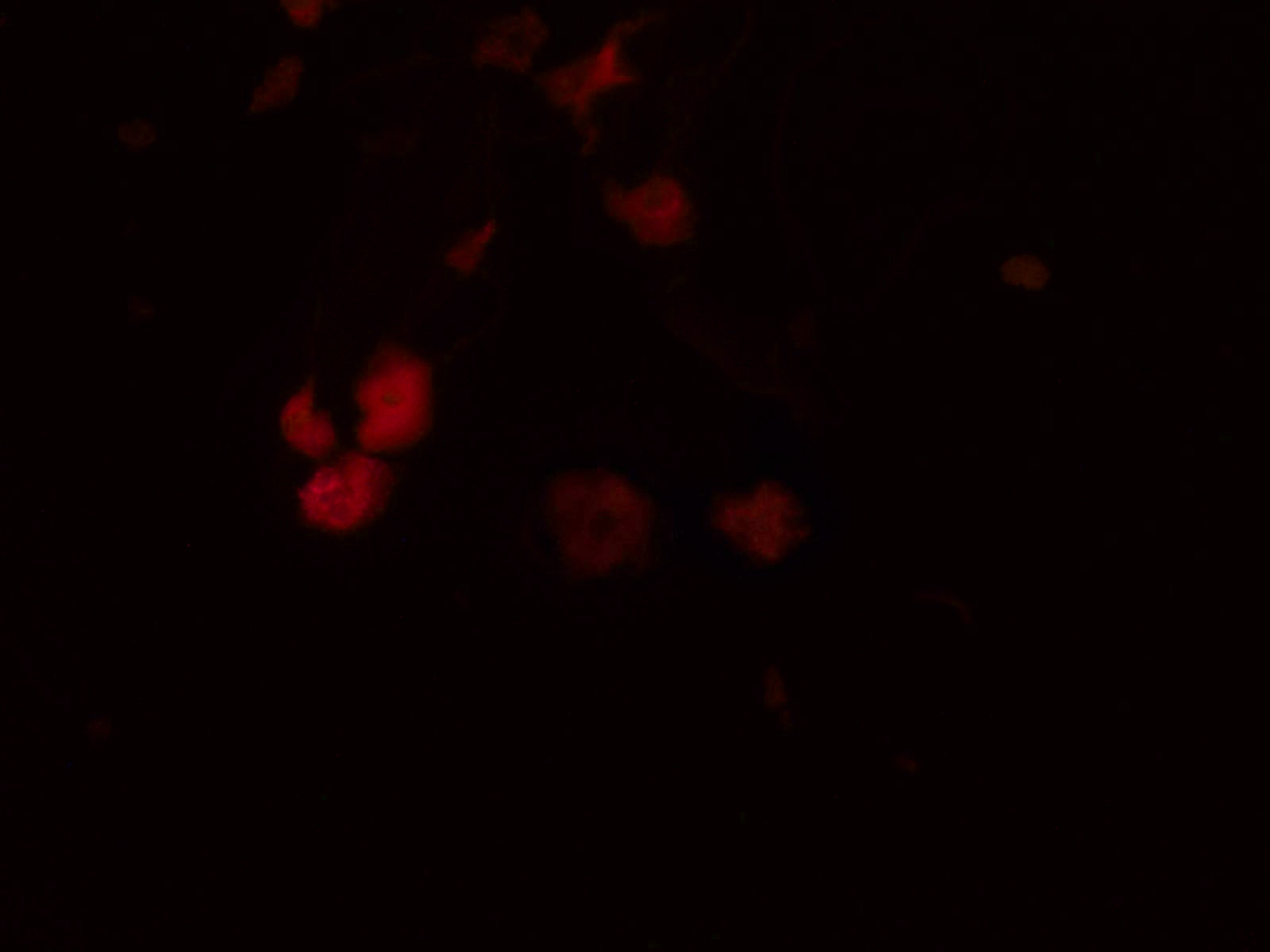

Supplement: Supplemental Information 10 [file peerj-07-6344-s010.zip › 72H Timp-3 20X.jpg]

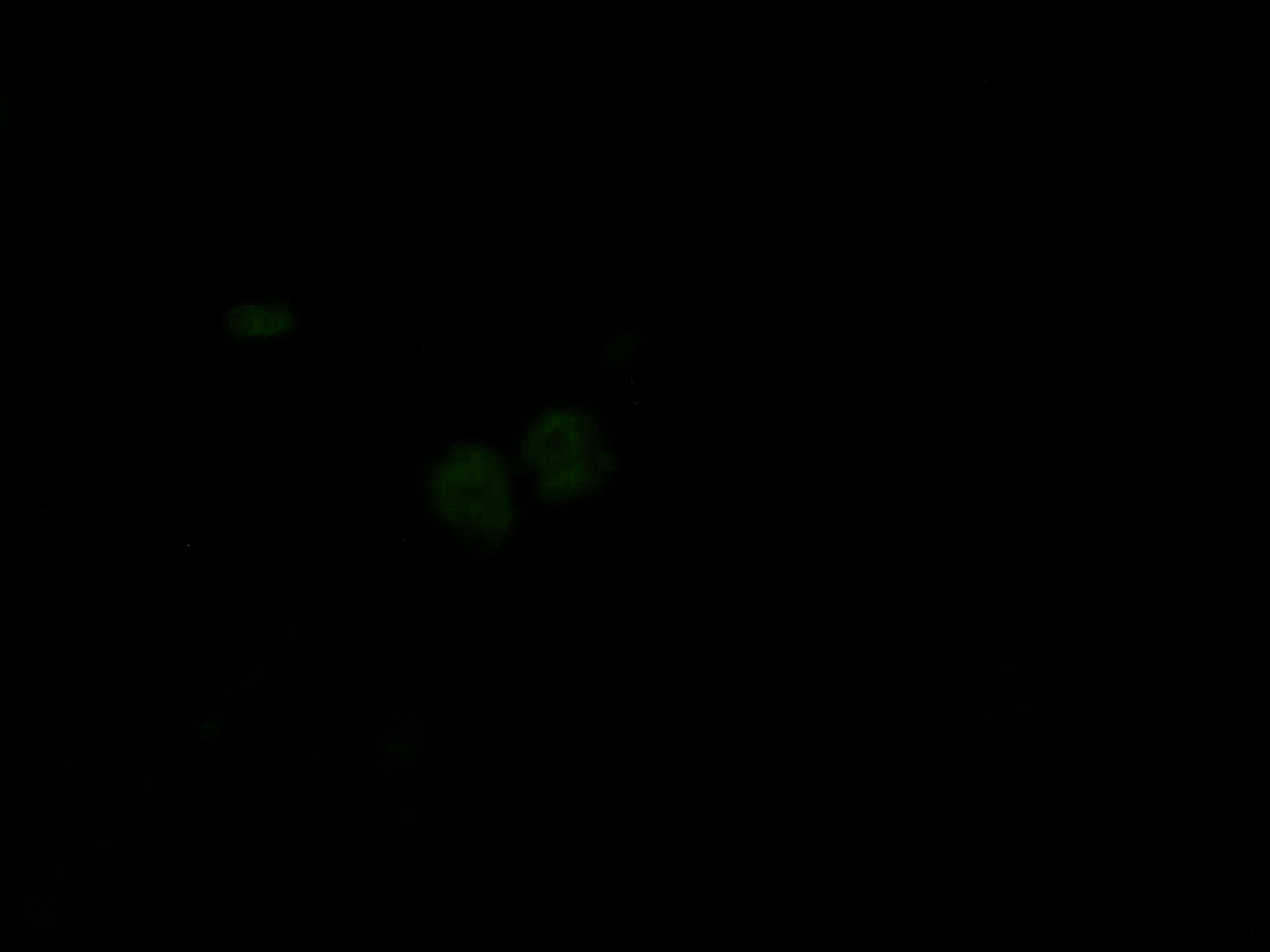

Supplement: Supplemental Information 10 [file peerj-07-6344-s010.zip › 96H Timp-2 20X.jpg]

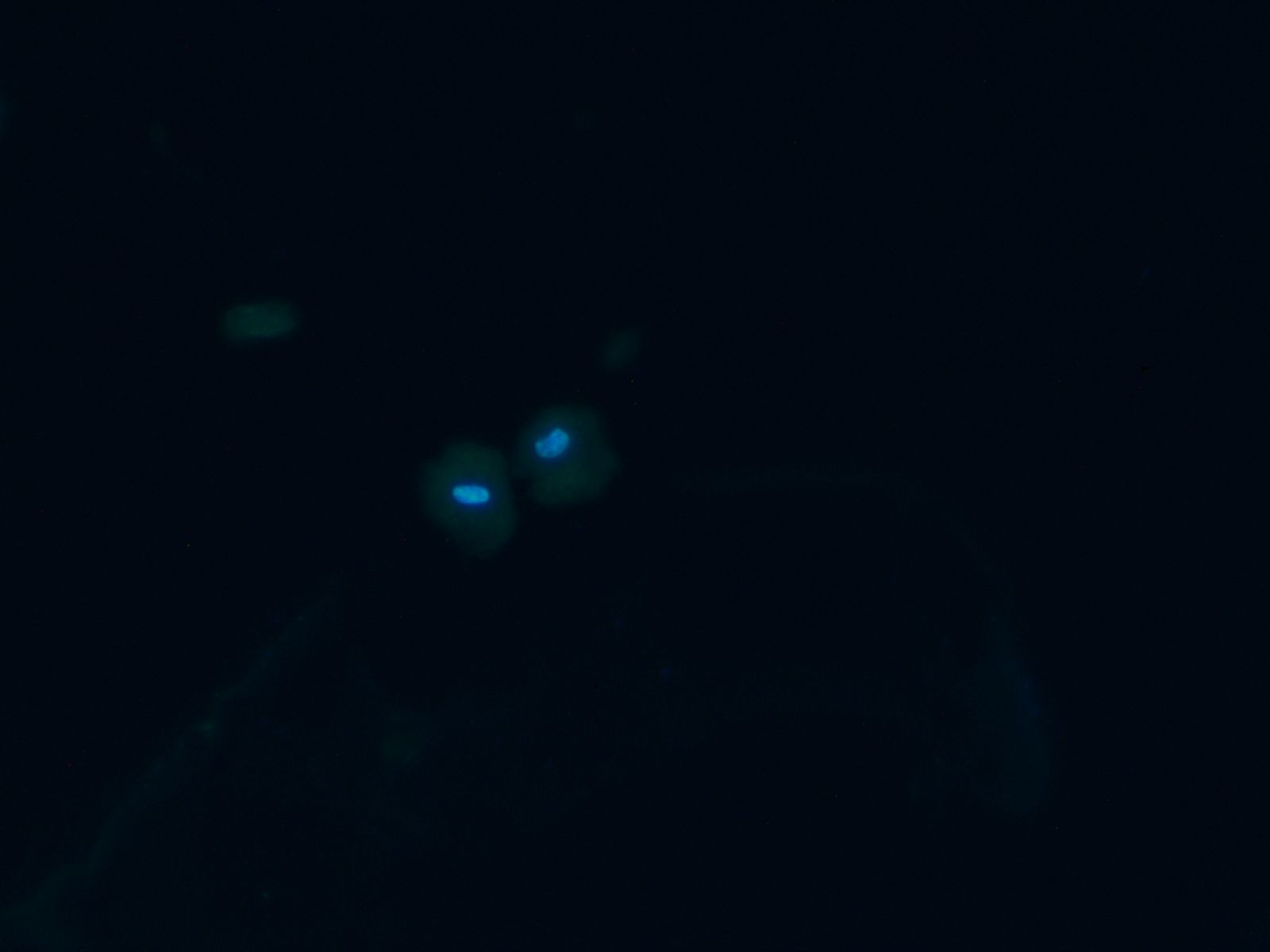

Supplement: Supplemental Information 10 [file peerj-07-6344-s010.zip › 96H Timp-23 20X(H).jpg]

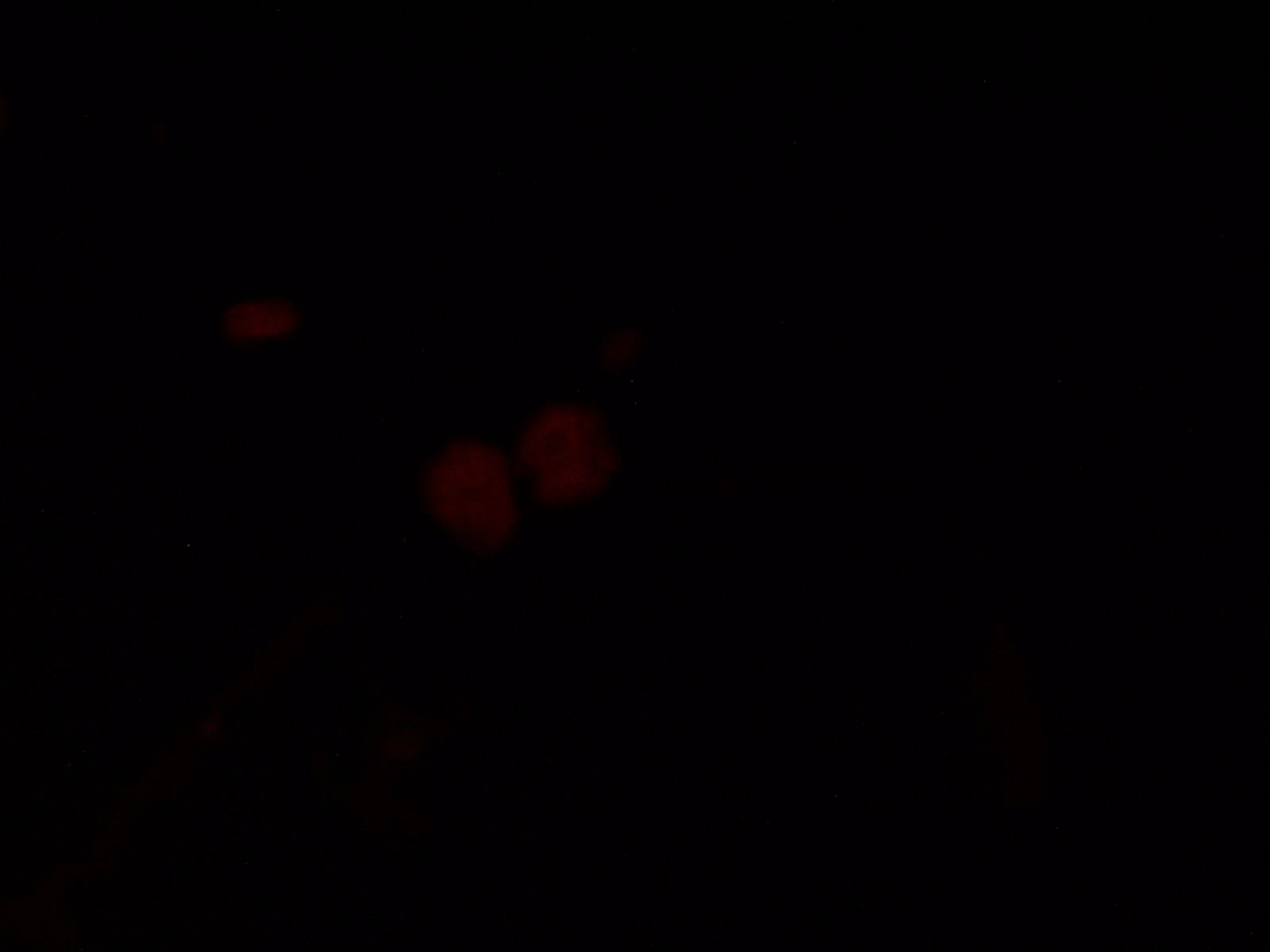

Supplement: Supplemental Information 10 [file peerj-07-6344-s010.zip › 96H Timp-3 20X.jpg]

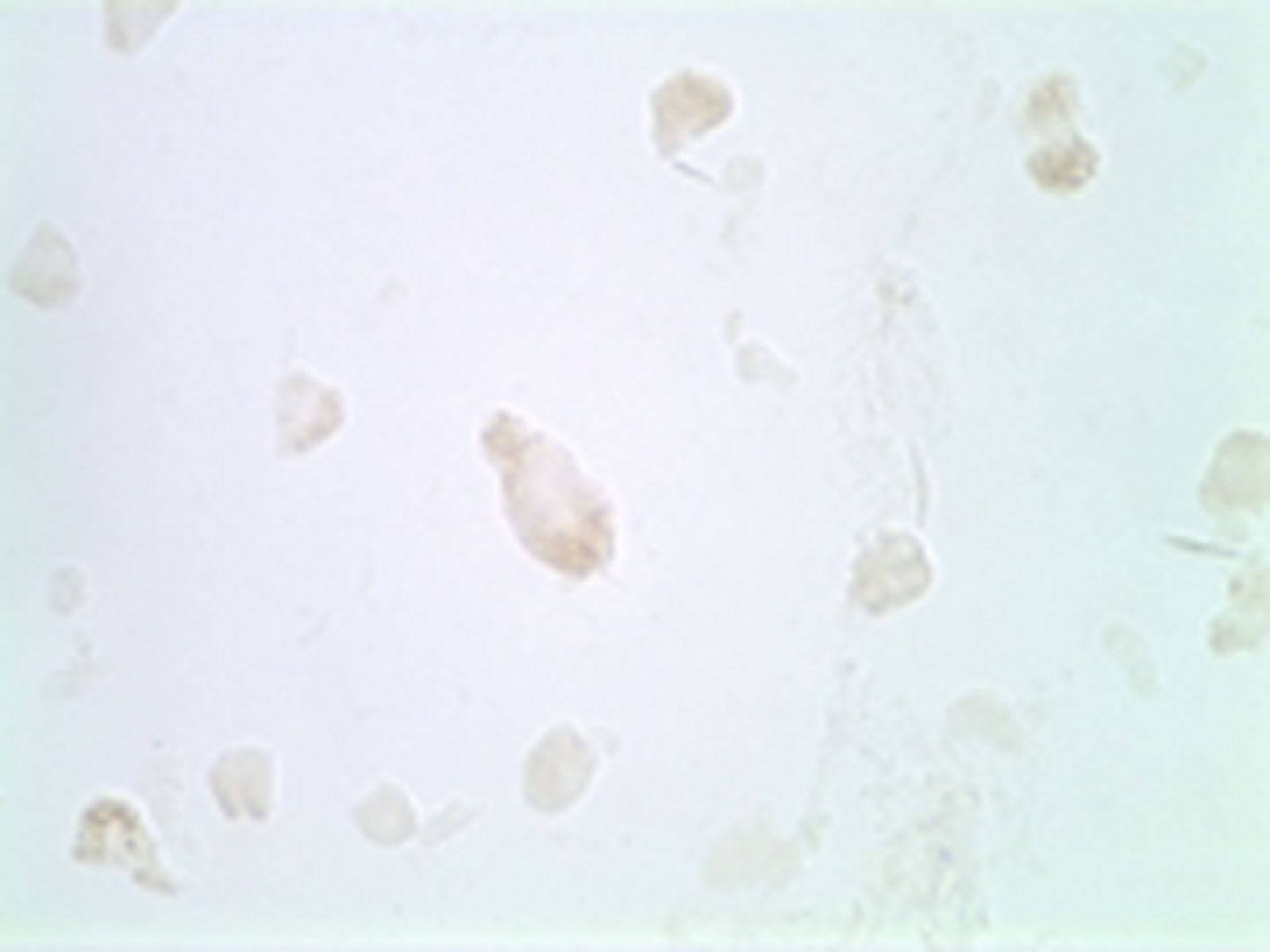

Supplement: Supplemental Information 11 [file peerj-07-6344-s011.zip › 24H-Casp&HSD20X(W).jpg]

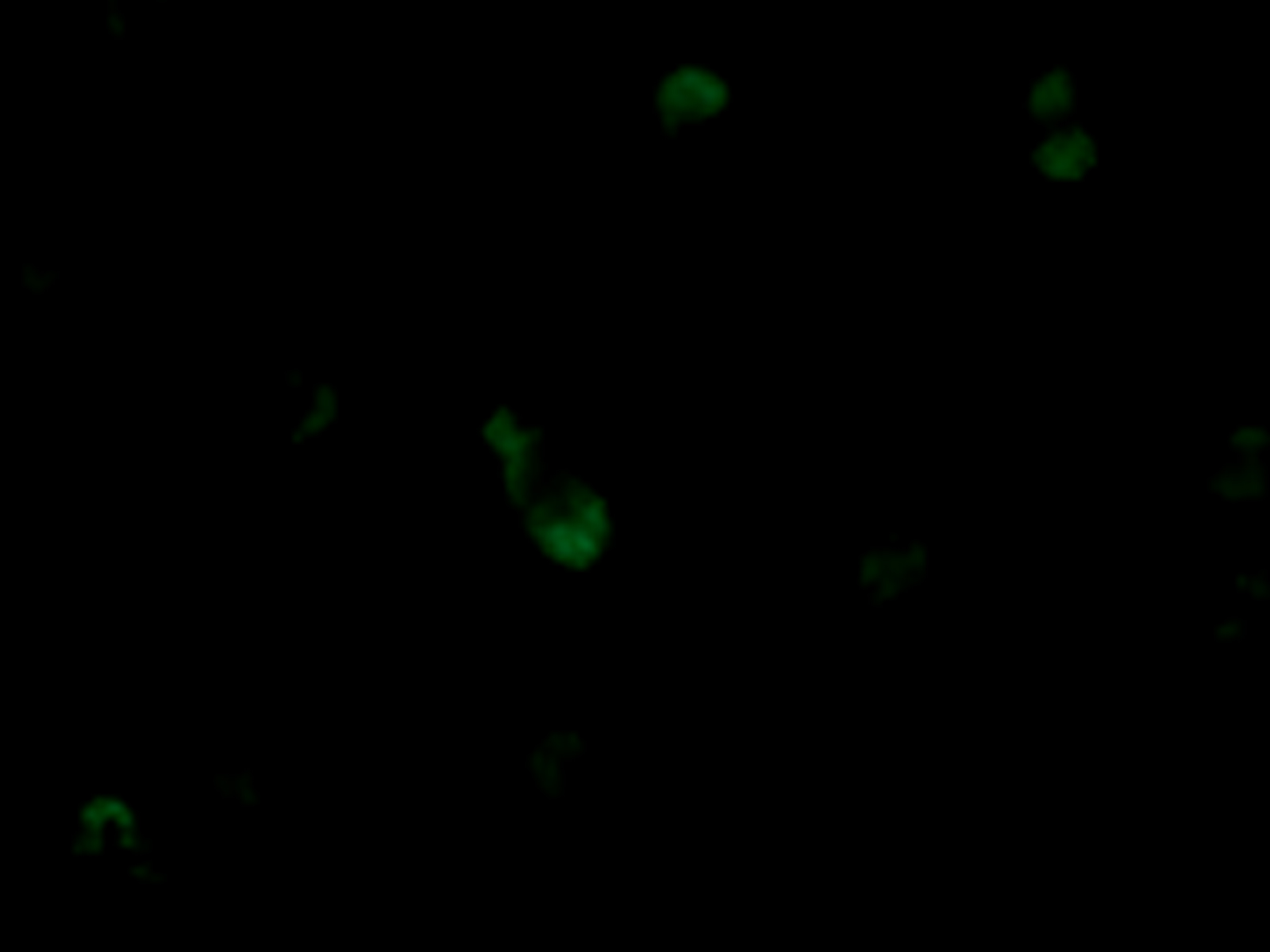

Supplement: Supplemental Information 11 [file peerj-07-6344-s011.zip › 24H-Casp20X(G).jpg]

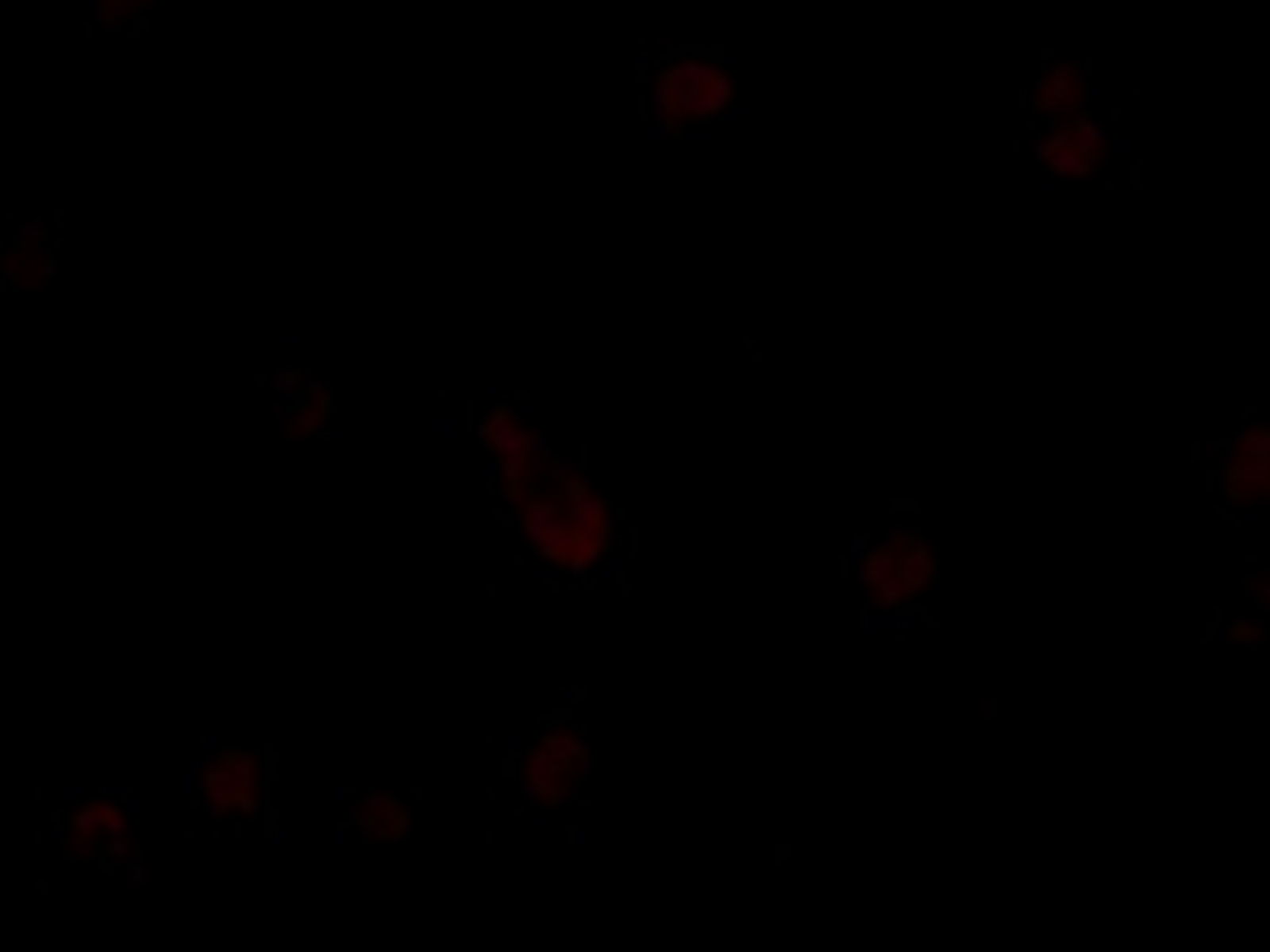

Supplement: Supplemental Information 11 [file peerj-07-6344-s011.zip › 24H-HSD20X(R).jpg]

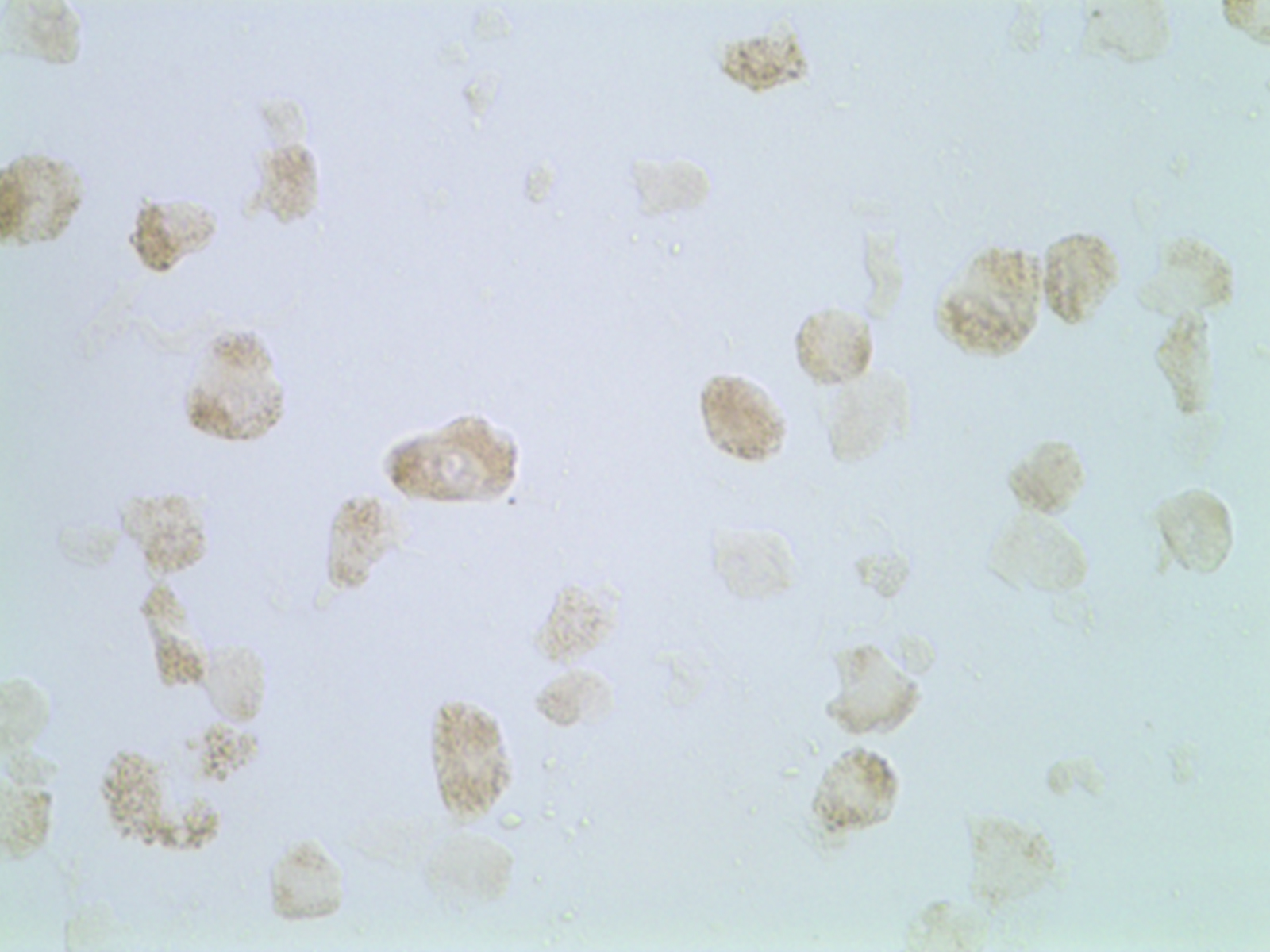

Supplement: Supplemental Information 11 [file peerj-07-6344-s011.zip › 96H-Casp&HSD20X(W).jpg]

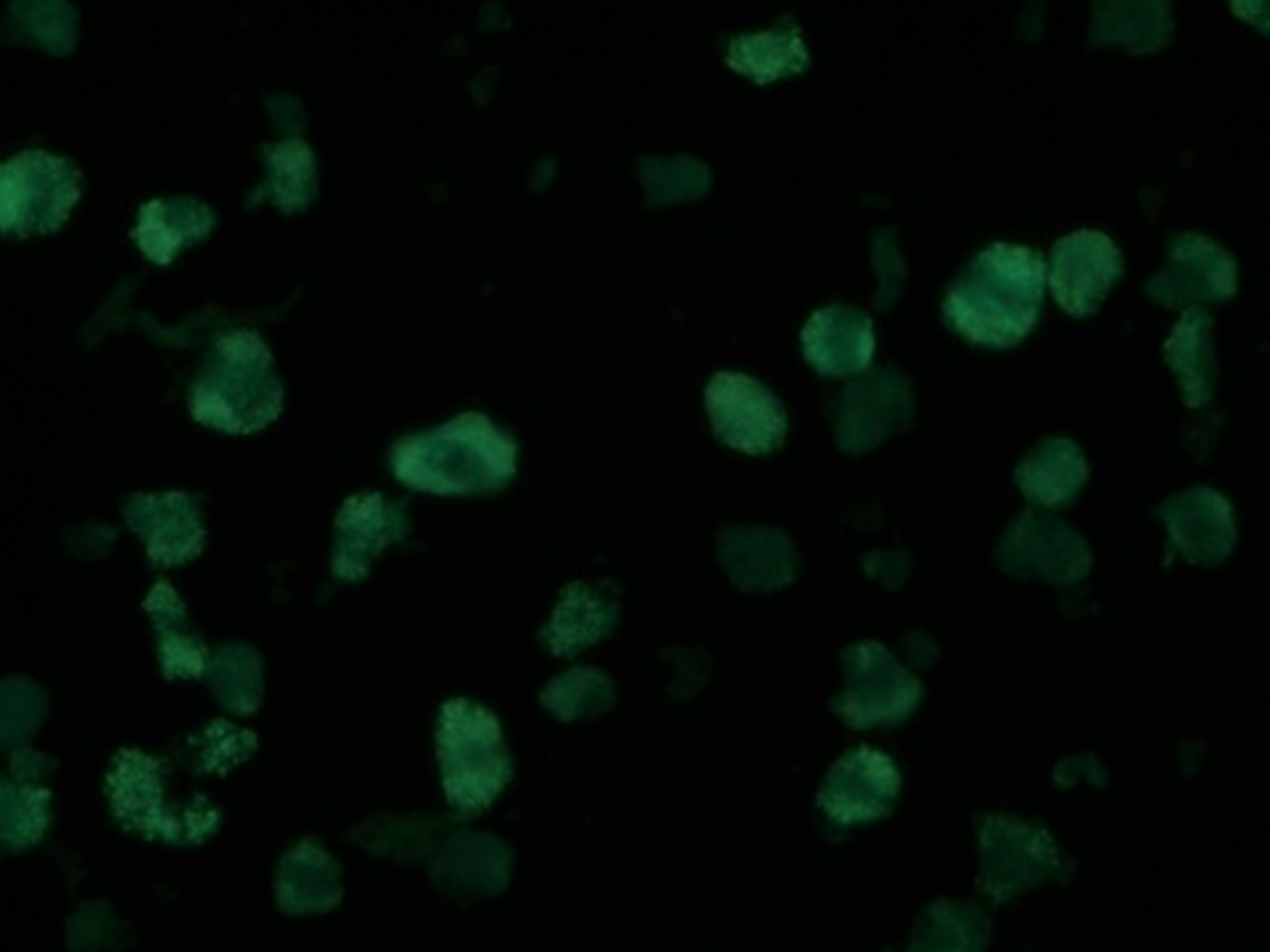

Supplement: Supplemental Information 11 [file peerj-07-6344-s011.zip › 96H-Casp20X(G).jpg]

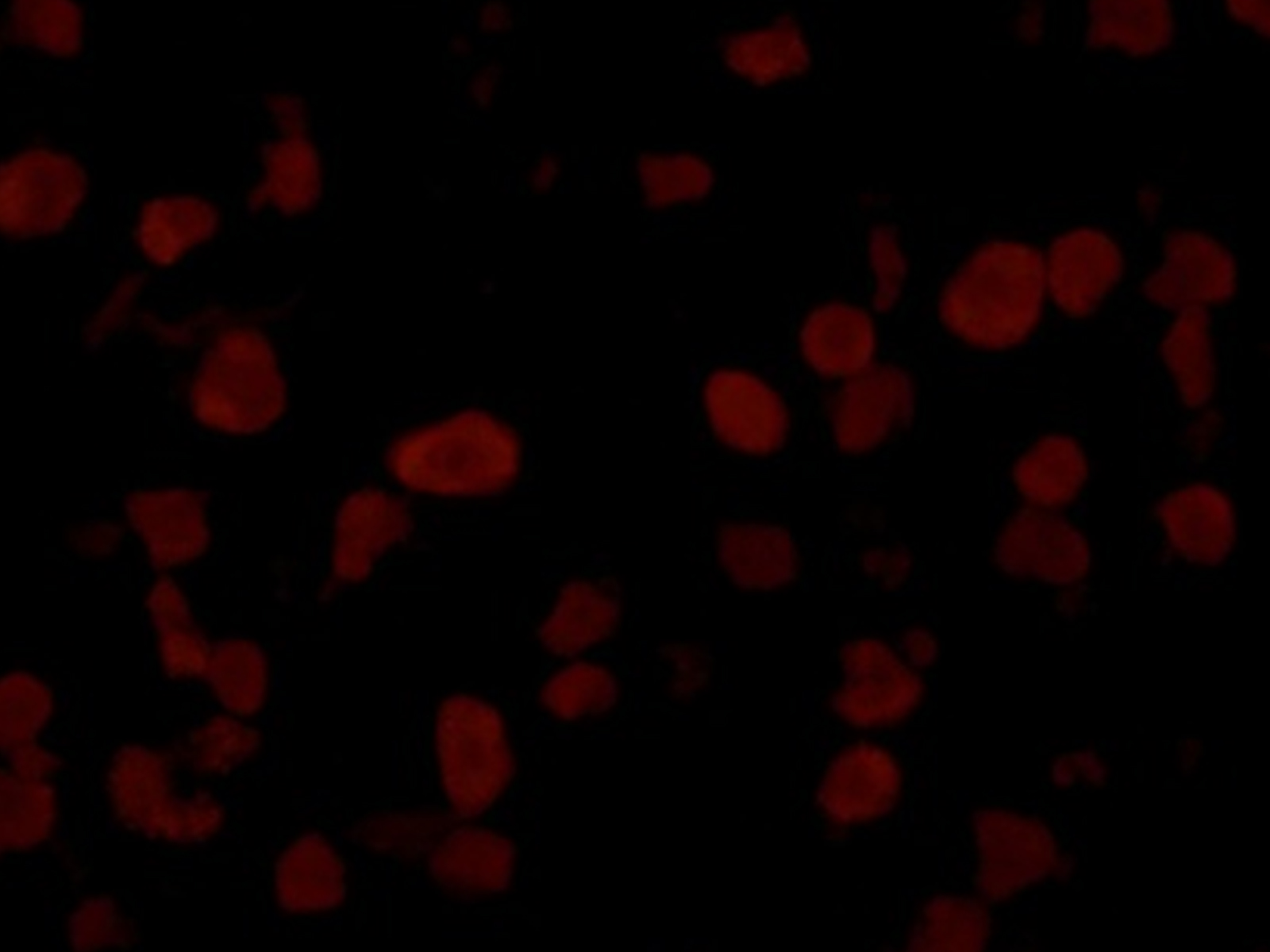

Supplement: Supplemental Information 11 [file peerj-07-6344-s011.zip › 96H-HSD20X(R).jpg]
